# Characterization of the genetic variation and evolutionary divergence of the CLEC18 family

**DOI:** 10.1186/s12929-024-01034-5

**Published:** 2024-05-20

**Authors:** Che-Mai Chang, Wei-Chiao Chang, Shie‐Liang Hsieh

**Affiliations:** 1 Genomics Research Center, Academia Sinica, No. 128, Sec. 2, Academia Rd., Nangang Dist., Taipei City, 115 Taiwan; 2 Department of Clinical Pharmacy, School of Pharmacy, Taipei Medical University, No.250, Wuxing St., Xinyi Dist, Taipei City, 110 Taiwan; 3 Master Program in Clinical Genomics and Proteomics, School of Pharmacy, Taipei Medical University, Taipei City, 110 Taiwan; 4 Department of Pharmacy, Wan Fang Hospital, Taipei Medical University, Taipei City, 116 Taiwan; 5 Integrative Research Center for Critical Care, Wan Fang Hospital, Taipei Medical University, Taipei City, 116 Taiwan; 6 Immunology Research Center, National Health Research Institutes, No. 35, Keyan Rd., Zhunan Township, Miaoli County, 350 Taiwan; 7 Institute of Clinical Medicine, National Yang Ming Chiao Tung University, Taipei City, 112 Taiwan; 8 Department of Medical Research, Taipei Veterans General Hospital, Taipei City, 112 Taiwan; 9 Department of Pharmacology, National Defense Medical Center, Taipei City, 114 Taiwan

**Keywords:** CLEC18, CAP/SCP/TAPS domain, CTLD, Long-read sequencing, Human pangenome, Single amino acid variant, Paralogous sequence variant, Polymorphic variant, Molecular evolution

## Abstract

**Background:**

The C-type lectin family 18 (CLEC18) with lipid and glycan binding capabilities is important to metabolic regulation and innate immune responses against viral infection. However, human CLEC18 comprises three paralogous genes with highly similar sequences, making it challenging to distinguish genetic variations, expression patterns, and biological functions of individual CLEC18 paralogs. Additionally, the evolutionary relationship between human CLEC18 and its counterparts in other species remains unclear.

**Methods:**

To identify the sequence variation and evolutionary divergence of human CLEC18 paralogs, we conducted a comprehensive analysis using various resources, including human and non-human primate reference genome assemblies, human pangenome assemblies, and long-read-based whole-genome and -transcriptome sequencing datasets.

**Results:**

We uncovered paralogous sequence variants (PSVs) and polymorphic variants (PVs) of human CLEC18 proteins, and identified distinct signatures specific to each CLEC18 paralog. Furthermore, we unveiled a novel segmental duplication for human CLEC18A gene. By comparing CLEC18 across human and non-human primates, our research showed that the CLEC18 paralogy probably occurred in the common ancestor of human and closely related non-human primates, and the lipid-binding CAP/SCP/TAPS domain of CLEC18 is more diverse than its glycan-binding CTLD. Moreover, we found that certain amino acids alterations at variant positions are exclusive to human CLEC18 paralogs.

**Conclusions:**

Our findings offer a comprehensive profiling of the intricate variations and evolutionary characteristics of human CLEC18.

**Supplementary Information:**

The online version contains supplementary material available at 10.1186/s12929-024-01034-5.

## Background

C-type lectins constitute a diverse group of proteins with an affinity for binding to carbohydrates. These proteins contain the carbohydrate recognition domain (CRD) or the C-type lectin-like domain (CTLD), enabling them to interact with a wide range of ligands in Ca^2+^-dependent or -independent manner. Within the extensive C-type lectin superfamily, there are 17 subgroups comprising over 1000 proteins, all of which play pivotal roles in immunity and maintaining internal balance [1, 2]. Among these subgroups, C-type lectin domain family 18 (CLEC18) stands out as a recently discovered cluster of C-type lectins classified under C-type lectin group XVI. CLEC18 proteins, encoded by CLEC18 genes, possess a distinctive combination of a cysteine-rich secretory protein/antigen 5/pathogenesis-related 1 (CAP) domain (also known as Tpx 1/antigen 5/pathogenesis related-1/Sc7 (TAPS) domain or sperm-coating glycoprotein (SCP) domain), a C-type lectin-like domain (CTLD), and epidermal growth factor (EGF) or EGF-like domains [3]. CLEC18 has been implicated in critical immune functions due to its strong binding affinity to various glycans such as fucoidan, β-glucan, and galactan. Several studies have suggested a close link between CLEC18 and innate immune responses to viral infections, including hepatitis B virus (HBV), hepatitis C virus (HCV), H5N1 influenza A virus (IAV), and dengue virus (DENV) [4–7]. In humans, the CLEC18 family comprises three members, namely CLEC18A, CLEC18B, and CLEC18C, which exhibit remarkably similar sequences [8]. In contrast, most other species possess only one CLEC18 gene [8]. This observation strongly suggests that human CLEC18 genes are paralogous and have evolved from genome duplication events. However, the relationship between human CLEC18 genes/proteins and their counterparts in other species has not yet been explored.

Apart from sequence homology, there is also genetic divergence and variation within human CLEC18 paralogs. Both paralogous sequence variants (PSVs) and polymorphic variants (PVs) have been identified within CLEC18A, CLEC18B, and CLEC18C. PSVs highlight distinctions between gene paralogs, while PVs reflect variations within specific CLEC18 members across populations. Recent studies have indicated that genetic variations leading to specific amino acid changes in CLEC18 may alter the binding abilities of CLEC18 proteins, potentially impacting human metabolic regulation [8, 9]. A single amino acid change from serine (S) to arginine (R) at position 339 within the CTLD of human CLEC18A has been shown to increase the binding affinity of toll-like receptor 3 (TLR3) and CLEC18A to poly(I:C), enhancing interferon (IFN) production against infection [6]. By screening human genome sequencing data among populations, the rs75776403, a missense variant that changes one amino acid residue from threonine (T) to methionine (M) in the CAP/SCP/TAPS domain of CLEC18A, was identified to be associated with human anthropometric, kidney, and hematological traits [9]. However, analyzing the genetic variations of individual CLEC18 genes remains challenging because distinguishing between the PSVs and PVs of CLEC18A, CLEC18B, and CLEC18C is intricate when using short-read sequencing technologies. The limited length of sequences generated by techniques like whole-genome sequencing (WGS), whole-exome sequencing (WES), or targeted sequencing can lead to misalignment to incorrect locations on the reference genome due to the substantial sequence similarity among human CLEC18 genes. Consequently, data from short-read sequencing techniques are insufficient for precisely identifying genetic variants within the CLEC18 genes.

Recent significant progress has been made by the T2T Consortium, which has comprehensively decoded the human genome using multiple long-read sequencing methods. This achievement has led to the development of a gapless human reference genome assembly called T2T-CHM13, which is invaluable for identifying complex genomic variations [10–12]. Concurrently, the Human Pangenome Reference Consortium (HPRC) has released the latest version of the human pangenome reference, containing 94 de novo phased whole genome assemblies from 47 individuals obtained through long-read sequencing [13]. In this study, we harnessed the T2T-CHM13 reference genome assembly, human pangenome assemblies, and additional long-read sequencing datasets to investigate previously reported and novel genetic variations within human CLEC18 protein sequences. Our aim was to provide a comprehensive characterization and differentiation of CLEC18A, CLEC18B, and CLEC18C. Additionally, we conducted systematic analyses to compare CLEC18 protein sequences and variations between humans and non-human primates. By employing this approach, our results offer an in-depth characterization and validation of the genomic variation and evolutionary divergence of CLEC18.

## Methods

### Data collection and selection

All data used in this study are publicly available and were obtained from the National Center for Biotechnology Information (NCBI) (https://www.ncbi.nlm.nih.gov), Ensembl (https://www.ensembl.org), and PacBio (https://www.pacb.com).

For comparisons between CLEC18 sequences in humans, both genome and protein sequences of the human *CLEC18A*, *CLEC18B*, and *CLEC18C* genes in the GRCh38.p14 and T2T-CHM13v2.0 reference assemblies were obtained from the NCBI gene database (https://www.ncbi.nlm.nih.gov/gene). In addition, genome sequences and gene annotations of 94 human *de novo* haplotype assemblies of 47 individuals, generated and released by the HPRC [13], were downloaded from the Ensembl project (https://projects.ensembl.org/hprc) for a comprehensive profiling of human CLEC18 protein sequence variants.

As for variant validation of human CLEC18, we additionally downloaded whole-genome sequencing (WGS) and whole-transcriptome sequencing (WTS) datasets, which were generated by the PacBio high-fidelity (HiFi) long-read sequencing platform, from PacBio (https://www.pacb.com/connect/datasets) and the NCBI Sequence Read Archive (SRA) database (https://www.ncbi.nlm.nih.gov/sra). As a result, we included three long-read WGS datasets (the GIAB trio HG002, HG003, and HG004) available in PacBio and another eight long-read WGS datasets in the NCBI SRA by searching “(pacbio HiFi) AND "homo sapiens "[Organism] AND "WGS"[Strategy]”. In addition, four long-read WTS datasets were obtained from the NCBI SRA by searching “(pacbio iso-seq) AND "homo sapiens"[Organism] AND "RNA-Seq"[Strategy]” for further validation.

As for sequence comparison across species, amino acid sequences of CLEC18 genes of humans and non-human primates were downloaded from the NCBI gene database (https://www.ncbi.nlm.nih.gov/gene) by searching “(c-type lectin domain family 18) AND "vertebrates"[porgn:__txid7742])) AND "primates"[porgn:__txid9443])”. As a result, we collected 224 protein sequences from 44 CLEC18/CLEC18-like genes, which were annotated with either “CLEC18” in name or with “C-type lectin domain family 18 member” in description, of humans and of 32 non-human primates.

### Multiple sequence alignments of the human CLEC18 gene and protein sequences

For human CLEC18 genes, multiple sequence alignments of CLEC18A, CLEC18B, and CLEC18C genomic sequences in human reference assemblies of GRCh38.p14 and T2T-CHM13v2.0 were carried out using Clustal Omega [14]. Additionally, we extracted upstream (10,000 bp) and downstream (9,000 or 10,000 bp) flanking sequences of the *CLEC18A* gene in the T2T-CHM13v2.0 reference assembly to discover and validate a structural variant of segmental duplication. As for human CLEC18 proteins, coding region sequences (CDSs) of CLEC18A, CLEC18B, and CLEC18C were obtained from the GRCh38.p14 and T2T-CHM13v2.0 reference assemblies. Genomic coordinates of CDSs of representative CLEC18 genes (RefSeq/MANE select) in GRCh38.p14 were used to infer CLEC18A, CLEC18B, and CLEC18C protein sequences in the T2T-CHM13v2.0 reference assembly. Multiple sequence alignments of human CLEC18 protein sequences were conducted using ClustalW [15].

### Identification of PSVs and PVs of human CLEC18 protein sequences by human pangenome references

In both the GRCh38.p14 and T2T-CHM13v2.0 human reference assemblies, it was observed that the CLEC18 gene cluster, containing *CLEC18A*, *CLEC18C*, and *CLEC18B*, is adjacent to *WW domain containing E3 ubiquitin protein ligase 2* (*WWP2*) and *golgi glycoprotein 1* (*GLG1*). *CLEC18A* and *CLEC18B* are respectively located downstream and upstream of *WWP2* and *GLG1* on chromosomes 16q22.1 and 16q23.1. *CLEC18C* is located on chromosome 16q22.1 between *CLEC18A* and *CLEC18B*. On the basis of such genomic characteristics, the entire CLEC18 gene cluster can be located by *WWP2* and *GLG1* in other genomes. Accordingly, we used *WWP2* and *GLG1* as adjacent markers to extract genomic regions containing the *CLEC18A*, *CLEC18C*, and *CLEC18B* genes in 94 human pangenome assemblies. The extracted genome sequences of pangenome assemblies were subsequently mapped to both the GRCh38.p14 and T2T-CHM13v2.0 reference assemblies using minimap2 (version 2.26-r1175) with a preset sequence divergence of 0.1% between target and reference assemblies [16]. After genome alignment, CDSs of CLEC18 genes in the pangenome assemblies were determined based on validated and inferred genomic coordinates of CDSs of the *CLEC18A*, *CLEC18B*, and *CLEC18C* genes in the GRCh38.p14 and T2T-CHM13v2.0 reference assemblies. We then determined the consensus CDS of each CLEC18 gene from sequences mapped to GRCh38.p14 and T2T-CHM13v2.0, and those with ambiguous (N) or incomplete (-) sequences were discarded in order to eliminate sequence ambiguity. Finally, CDSs of CLEC18A, CLEC18B, and CLEC18C in pangenome assemblies were translated from nucleic acid sequences into amino acid sequences. For these CLEC18 protein sequences, only those with 446 amino acids and without a truncating stop codon (*) were included. As a result, 90 CLEC18A, 91 CLEC18B, and 89 CLEC18C protein sequences from 94 human pangenome assemblies remained.

Protein sequences of CLEC18A, CLEC18B, and CLEC18C from pangenome assemblies were further aligned to each other using ClustalW [15]. Based on multiple sequence alignments, single amino acid variants (SAVs) across different CLEC18 protein sequences and different assemblies were determined. These SAVs were then classified into PSVs and PVs according to whether the SAV was caused by the difference between CLEC18 paralogs or by genetic polymorphism between individuals. Therefore, a SAV with different dominant amino acids at paralogous positions of CLEC18A, CLEC18B, and CLEC18C protein sequences was determined to be a PSV; otherwise, it was defined as a PV.

After variant calling, amino acid combinations of PSVs or PSVs+PVs were further identified for each CLEC18 protein sequence of the pangenome assemblies. In addition, whether a CLEC18A segmental duplication existed was examined by genome/assembly alignment to the *CLEC18A* locus in the T2T-CHM13v2.0 reference assembly.

### Profiling of PSVs and PVs of human CLEC18 protein sequences in long-read WGS datasets

Public PacBio HiFi long-read WGS datasets were directly aligned to both NCBI GRCh38.p14 and T2T-CHM13v2.0 reference assemblies using pbmm2 (version 1.9.0 by Pacific Biosciences) with a preset alignment mode of HiFi. After mapping long reads, CLEC18-derived alignments that overlapped genomic regions of the *CLEC18A*, *CLEC18B*, and *CLEC18C* genes (69943519-69966623, 74408631-74424249, and 70173787-70186895 on chromosome 16 in GRCh38.p14; 75749365-75777910, 80223868-80242200, and 75983920-75997950 on chromosome 16 in T2T-CHM13v2.0) were extracted using Samtools (version 1.15.1) [17]. PSVs and PVs, determined from pangenome assemblies, on CLEC18-derived alignments were identified based on their GRCh38.p14 and/or T2T-CHM13v2.0 coordinates. Nucleic acid sequences of PSVs and PVs on CLEC18-derived alignments were subsequently determined and translated into amino acids. As a result, amino acid combinations of seven PSVs and 12 common PVs (frequencies of all CLEC18 paralogs were > 0.01) of CLEC18-derived sequences were obtained. Finally, only those CLEC18-derived sequences in which amino acids of 19 PSVs/PVs could be successfully deciphered were included.

For validation of CLEC18A segmental duplication, long-read WGS alignments against to the *CLEC18A* locus in the T2T-CHM13v2.0 reference assembly were examined.

### Profiling of PSVs and PVs of human CLEC18 protein sequences in long-read WTS datasets

Public long-read WTS datasets generated by the PacBio Iso-Seq approach were analyzed using recommended bulk Iso-Seq workflow (https://isoseq.how/). In brief, iso-seq HiFi reads were aligned to the reference assembly of GENCODE release 43 (GRCh38.p13) and subsequently processed with collapsing, sorting, classification, and filtering steps. Sequence alignment was performed using pbmm2 (version 1.9.0 by Pacific Biosciences) with a preset alignment mode of ISOSEQ. To collapse redundant transcripts, isoseq3 (version 3.8.1 by Pacific Biosciences) was used. The sorting, classification, and filtering of transcripts were conducted using pigeon (version 1.0.0 by Pacific Biosciences). We then extracted CLEC18-derived alignments that were classified as CLEC18A, CLEC18B, or CLEC18C isoforms. Identifying amino acid combinations of PSVs and PVs of CLEC18-derived sequences was conducted as described above. Similarly, only CLEC18-derived sequences with non-missing amino acids at 19 PSV/PV positions were included.

### Multiple sequence alignments of CLEC18 protein sequences across species

Before conducting multiple sequence alignments, the 224 originally downloaded CLEC18/CLEC18-like protein sequences of humans and non-human primates were filtered through several steps. We first selected three CLEC18 protein sequences, which are curated (NP_*) and annotated with “RefSeq select” or “MANE select” in the GRCh38.p14 human reference assembly, as representatives of human CLEC18A, CLEC18B, and CLEC18C protein sequences. Since all of these representative CLEC18 protein sequences in humans possessed 446 amino acids, we applied 446 as a general CLEC18 protein length to filter CLEC18/CLEC18-like protein sequences of non-human primates, which were based on computational predictions with highly diverse lengths and had not been curated/validated (XP_*) in the NBCI database. Accordingly, only CLEC18/CLEC18-like protein sequences with lengths from 444 to 448 amino acids (446 ± 0.5%) in non-human primates were included. As a result, 64 CLEC18/CLEC18-like protein sequences encoded by 31 CLEC18/CLEC18-like genes of 25 non-human primates and three human CLEC18 protein sequences remained after the filtering steps. For non-human primates, we determined the consensus CLEC18/CLEC18-like protein sequence of each CLEC18/CLEC18-like gene by sequence alignment using ClustalW [15]. The minimum frequency threshold for the agreement of consensus (or dominant) amino acids at each position of CLEC18 protein sequences from the same CLEC18/CLEC18-like gene was set to 0.5. Finally, we obtained 34 CLEC18/CLEC18-like representative protein sequences for humans and 25 non-human primates. Herein, the non-human primates included Ma's night monkey (*Aotus nancymaae*), white-tufted-ear marmoset (*Callithrix jacchus*), Panamanian white-faced capuchin (*Cebus imitator*), sooty mangabey (*Cercocebus atys*), green monkey (*Chlorocebus sabaeus*), Angola colobus (*Colobus angolensis palliatus*), western lowland gorilla (*Gorilla gorilla gorilla*), silvery gibbon (*Hylobates moloch*), crab-eating macaque (*Macaca fascicularis*), rhesus monkey (*Macaca mulatta*), pig-tailed macaque (*Macaca nemestrina*), Tibetan macaque (*Macaca thibetana thibetana*), drill (*Mandrillus leucophaeus*), northern white-cheeked gibbon (*Nomascus leucogenys*), pygmy chimpanzee (*Pan paniscus*), chimpanzee (*Pan troglodytes*), olive baboon (*Papio anubis*), Ugandan red colobus (*Piliocolobus tephrosceles*), Sumatran orangutan (*Pongo abelii*), Bornean orangutan (*Pongo pygmaeus*), black snub-nosed monkey (*Rhinopithecus bieti*), golden snub-nosed monkey (*Rhinopithecus roxellana*), Bolivian squirrel monkey (*Saimiri boliviensis*), siamang (*Symphalangus syndactylus*), and Francois's langur (*Trachypithecus francoisi*).

Multiple sequence alignments of these CLEC18/CLEC18-like representative protein sequences across humans and non-human primates were conducted using ClustalW [15]. The gappy sites with missing rates more than 90% across the CLEC18/CLEC18-like protein sequences were removed. Conservation of CLEC18/CLEC18-like protein sequences across species was determined by the frequency of dominant amino acids at each position of the alignment. The location of the CAP/SCP/TAPS domain (a.a.45-190), EGF domain (a.a.232-261), EGF-like domain (a.a.264-292), and CTLD (a.a.298-433) of the CLEC18/CLEC18-like protein sequence alignment was determined by reference to positions of these domains on human CLEC18A protein sequence according to the NCBI gene database and a previous study [8].

### Phylogenetic analysis of CLEC18 protein sequences across species

Pairwise distances from aligned CLEC18/CLEC18-like representative protein sequences of humans and non-human primates were calculated to generate a sequence identity matrix. The matrix was subsequently applied to phylogenetic reconstruction using the BIONJ algorithm [18]. A phylogenetic tree was then built to interrogate phylogenetic relationships between CLEC18/CLEC18-like proteins of different species. Both human reference and pangenome-consensus CLEC18A, CLEC18B, and CLEC18C protein sequences were compared to CLEC18/CLEC18-like protein sequences of non-human primates by the phylogenetic analysis.

### Data analysis and visualization

All genomic and sequencing data were analyzed on a Unix-like system. Data were visualized using R software (version 4.1.2 or 4.2.0). Multiple sequence alignments were performed using the *msa* R package. Graphics of the phylogenetic tree, multiple protein sequence alignments, sequence conservation, and protein domain architecture were generated using the *ggplot2*, *ggtree*, and *ggmsa* R packages. Multiple gene sequence alignment was illustrated using the *ggplot2* and *seqvisr* R packages. Logo plots were produced using the *ggplot2* and *ggseqlogo* R packages. Sequence alignment of the segmental duplication of the *CLEC18A* gene was visualized using the *Gviz* and *grDevices* R packages.

## Results

### Data source and study design for the variation and phylogeny of CLEC18

**Fig. 1:**
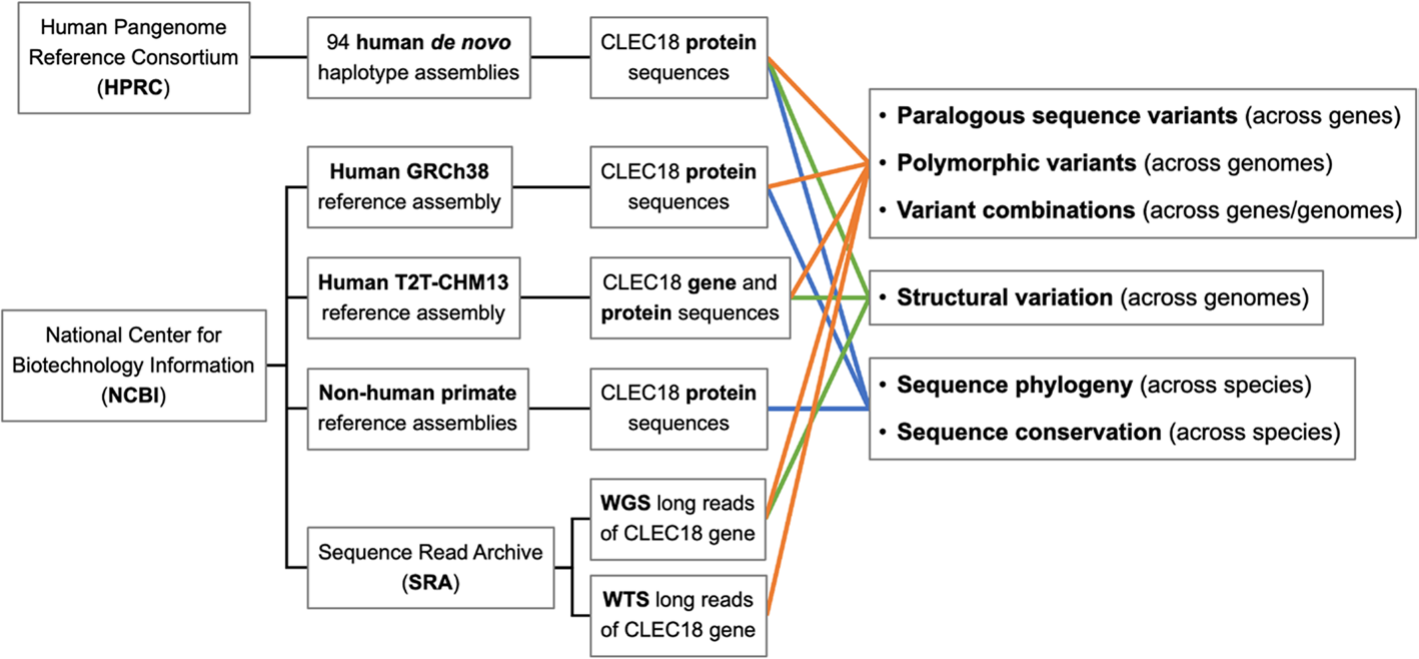
Study design and workflow. Reference CLEC18 gene and protein sequences were obtained from reference genome assemblies of humans and non-human primates in the National Center for Biotechnology Information (NCBI). Whole-genome/transcriptome long-read sequencing (WGS/WTS) data were downloaded from the Sequence Read Archive (SRA) of the NCBI and subjected to alignment to extract long reads mapped to CLEC18 genes. Population CLEC18 protein sequences were obtained by analyzing 94 human *de novo* haplotype assemblies released by the Human Pangenome Reference Consortium (HPRC). These CLEC18 gene and protein sequences were analyzed to profile CLEC18 genetic variants across CLEC18 genes and across different genomes, to identify CLEC18 structural variation across different genomes, and to investigate CLEC18 protein sequence phylogeny and conservation across species

We conducted variant identification and phylogenetic analysis for CLEC18 gene and protein sequences using data from various sources (see Fig. 1). To pinpoint amino acid variants presented in the different human CLEC18 paralogs (CLEC18A, CLEC18B, and CLEC18C), we gathered their protein sequences from the reference genome database, human pangenome datasets, and the high-throughput sequencing repository. For the identification of structural variations within human CLEC18 genes, we obtained the sequences of these genes from an alternate assembly of the reference genome, human pangenome datasets, and the high-throughput sequencing data. Furthermore, we sourced CLEC18 protein sequences from both humans and non-human primates from human pangenome datasets and the reference genome database.

### Identification of variations in CLEC18 paralogs by human reference genome assemblies

To explore distinctions among human CLEC18 proteins, we compared the amino acid sequences of CLEC18A, CLEC18B, and CLEC18C in the human reference genomes. In this endeavor, we harnessed the newly released human reference genome assembly, T2T-CHM13, based on long-read sequencing technologies that offer enhanced sequence continuity and the ability to resolve complex structural variations. This approach allowed us to discover and validate sequence variants between human CLEC18 proteins.

**Fig. 2:**
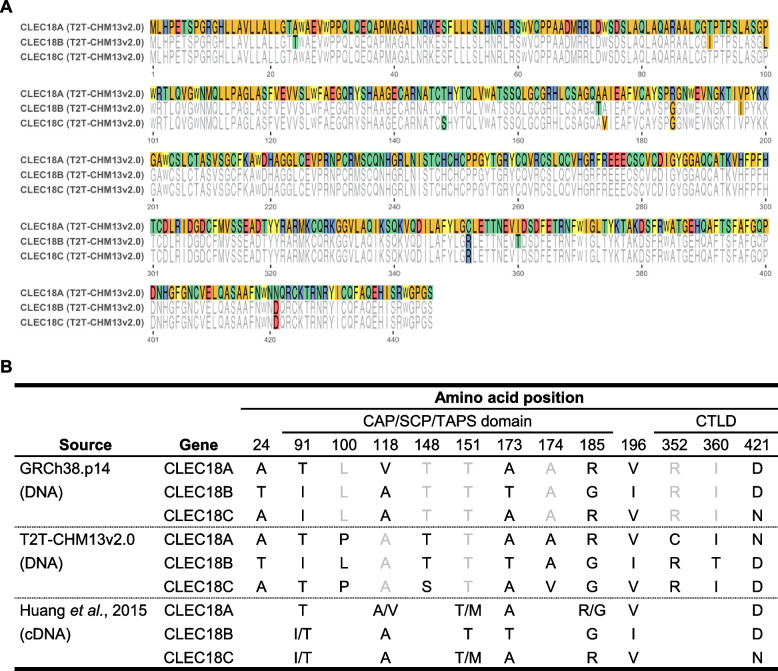
Identification of paralogous sequence variants (PSVs) of CLEC18 protein sequences in the T2T-CHM13v2.0 human reference genome assembly. **A** Comparison of human CLEC18A, CLEC18B, and CLEC18C protein sequences from the T2T-CHM13v2.0 assembly by multiple sequence alignments is shown. The protein sequence of CLEC18A was used as a reference for multiple sequence comparisons. Amino acid changes among CLEC18 protein sequences were determined as PSVs and highlighted by different colors which represent physiochemical properties of distinct amino acids. **B** PSVs between human CLEC18 protein sequences based on the GRCh38.p14 assembly, T2T-CHM13v2.0 assembly, and a previous study [8] are summarized

By aligning CLEC18A, CLEC18B, and CLEC18C protein sequences predicted from the T2T-CHM13v2.0 reference assembly, we identified 11 single amino acid variants (SAVs) (see Fig. 2A). Furthermore, when we compared CLEC18 amino acid sequences between the T2T-CHM13v2.0 and GRCh38.p14 reference assemblies, we detected 12 SAVs among CLEC18A, CLEC18B, and CLEC18C protein sequences (as shown in Fig. S1). We combined these results with those from a previous study [8] that reported 8 SAVs on the CAP/SCP/TAPS domain and CTLD of CLEC18 proteins through genotyping and sequencing of human CLEC18 cDNA. Consequently, we identified 13 SAVs classified as PSVs, indicating differences in amino acid sequences among CLEC18 protein paralogs in humans (refer to Fig. 2B). Of these PSVs, eight (located at amino acid positions 91, 100, 118, 148, 151, 173, 174, and 185) are situated within the CAP/SCP/TAPS domain, three (at positions 352, 360, and 421) within the CTLD, and two (at positions 24 and 196) in non-domain regions of CLEC18 proteins. Notably, five of these PSVs (at positions 100, 148, 174, 352, and 360) were exclusively found in the T2T-CHM13v2.0 assembly. Conversely, another six PSVs (at positions 24, 91, 173, 185, 196, and 421) were present in both the GRCh38.p14 and T2T-CHM13v2.0 assemblies, with most of them (except position 24) also reported in Huang *et al.*'s study. However, three of these PSVs (at positions 24, 173, and 196) exhibited consistent patterns of amino acid changes across CLEC18 protein paralogs in both reference assemblies and Huang *et al.*'s study, whereas the other three (at positions 91, 185, and 421) showed inconsistent patterns of amino acid alterations between the GRCh38.p14 assembly, the T2T-CHM13v2.0 assembly, and Huang *et al.*'s study. Furthermore, one PSV (at position 118) was identified in the GRCh38.p14 assembly and Huang *et al.*'s study but not in the T2T-CHM13v2.0 assembly.

Additionally, we observed that the last PSV (at position 151) was exclusively reported in Huang *et al.*'s study. These results underscore the complexity of PSVs in human CLEC18 protein paralogs and the challenges associated with distinguishing between CLEC18A, CLEC18B, and CLEC18C proteins based solely on their sequences. Nonetheless, the long-read-based T2T-CHM13v2.0 assembly proved invaluable for uncovering and validating PSVs in human CLEC18 protein paralogs.

**Fig. 3:**
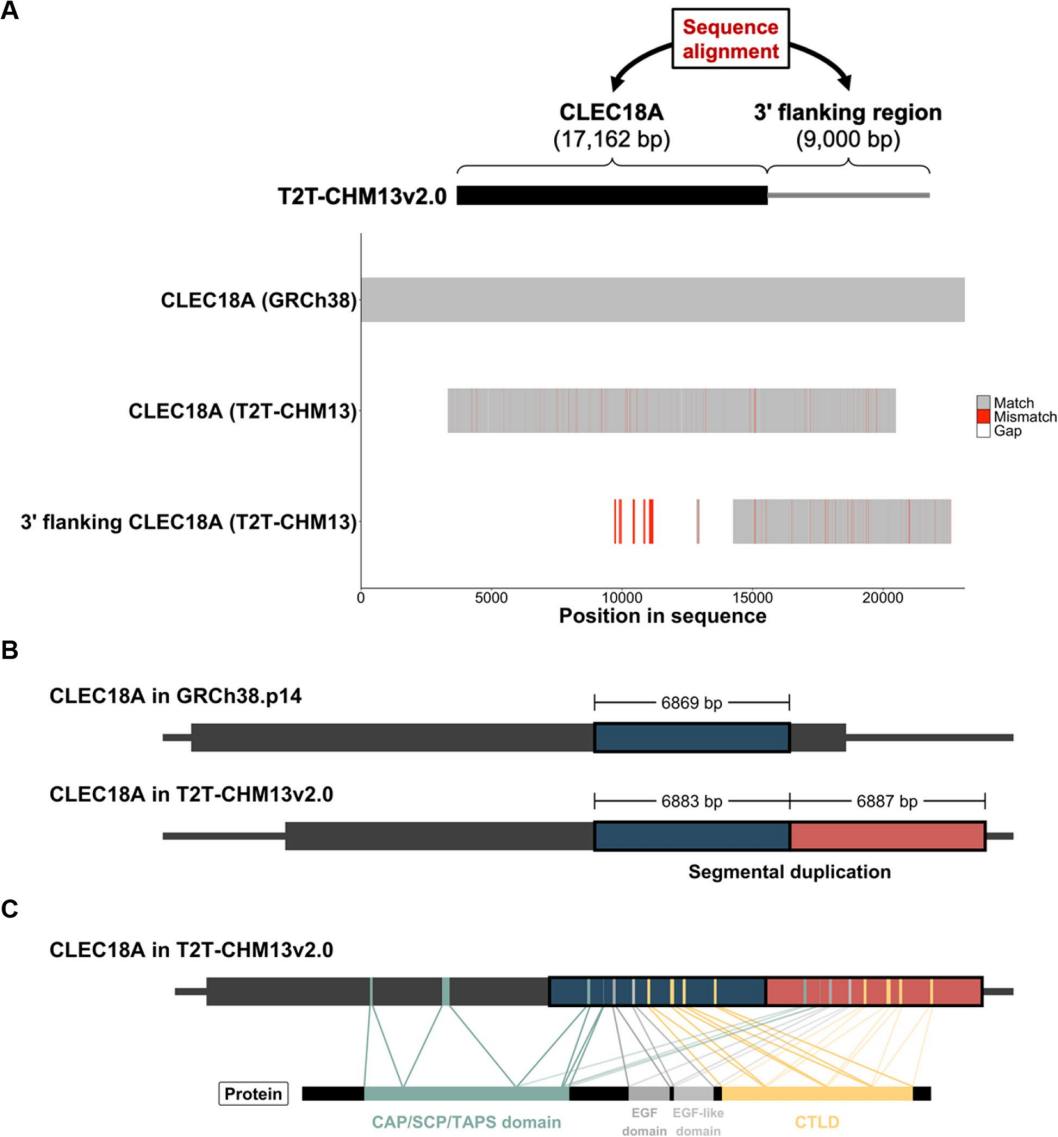
Identification of segmental duplication of the *CLEC18A* gene in the T2T-CHM13v2.0 human reference genome assembly. **A** Schematic diagram illustrating how the DNA sequence of the *CLEC18A* gene was compared to the sequence of its 3’ flanking region (9,000 bp) in T2T-CHM13v2.0 (upper panel). Comparison of DNA sequences between the *CLEC18A* gene in GRCh38.p14 and the *CLEC18A* gene in T2T-CHM13v2.0 and the 3’ flanking region of the *CLEC18A* gene in T2T-CHM13v2.0 was performed by a multiple sequence alignment (lower panel). **B** Schematic diagram illustrating a duplicated segment (colored red) located downstream of the *CLEC18A* gene in the T2T-CHM13v2.0 assembly. The region of the *CLEC18* gene (colored blue) highly identical to the segmental duplication in the GRCh38.p14 and T2T-CHM13v2.0 assemblies is shown. Lengths of these regions in the GRCh38.p14 and T2T-CHM13v2.0 assemblies are labeled. **C** Schematic diagram illustrating the connections between the genomic ranges of exons (top) and the corresponding protein regions (bottom) of the CAP/SCP/TAPS domain, EGF domain, EGF-like domain, and CTLD of duplicated segments of CLEC18A

In addition to PSVs in CLEC18, we identified a structural variation in the CLEC18A locus within the T2T-CHM13v2.0 reference assembly. Initially, we compared DNA sequences of the CLEC18A gene in the GRCh38.p14 and T2T-CHM13v2.0 assemblies and noted a high degree of sequence identity (as shown in Fig. S2A). Interestingly, the CLEC18A gene in the T2T-CHM13v2.0 assembly was observed to be shorter than that in the GRCh38.p14 assembly. Consequently, we incorporated an extended DNA sequence that covered the CLEC18A gene body along with its 5' and 3' flanking regions (with extensions of 10,000 bp at both ends) from the T2T-CHM13v2.0 assembly for further analysis. When comparing the DNA sequences of the CLEC18A gene in the GRCh38.p14 assembly and the extended CLEC18A gene sequence from the T2T-CHM13v2.0 assembly, we observed substantial overlap. However, an alignment gap within the CLEC18A gene sequence from the GRCh38.p14 assembly was evident (as depicted in Fig. S2B). Upon further sequence alignments, we noticed that a portion of the CLEC18A gene sequence from the GRCh38.p14 assembly exhibited high similarity to both the 3' end and the 3' flanking region of the CLEC18A gene sequence from the T2T-CHM13v2.0 assembly (see Fig. S2A, B). Subsequently, we compared the entire CLEC18A gene sequence (17,162 bp) and its 3' flanking genomic sequence (9,000 bp) and identified a significant overlapping region (refer to Fig. 3A). This region, which spans 6,869 bp in the CLEC18A gene in the GRCh38.p14 assembly, is duplicated as 6,883 and 6,887 bp segments in the T2T-CHM13v2.0 assembly (see Fig. 3B). This segmental duplication results in duplicated exon sequences of the CLEC18A gene, corresponding to amino acid sequences of partial CAP/SCP/TAPS domain, EGF domain, EGF-like domain, and CTLD of the CLEC18A protein in the T2T-CHM13 genome (illustrated in Fig. 3C).

### Comprehensive exploration of variations in CLEC18 paralogs by human pangenome assemblies

From the T2T-CHM13v2.0 reference assembly, we uncovered additional PSVs within CLEC18 proteins and identified a novel segmental duplication in the CLEC18A locus. Nevertheless, we observed inconsistent patterns of paralogous variation between the GRCh38.p14 and T2T-CHM13v2.0 reference assemblies, which might be attributed to genetic polymorphism within reference genomes. To comprehensively profile PSVs across human CLEC18 protein paralogs, we conducted an in-depth analysis of CLEC18 loci within human pangenome assemblies generated from long-read sequencing data by the Human Pangenome Reference Consortium (HPRC).

Initially, we extracted genomic regions containing the CLEC18 loci, situated between the WW domain-containing E3 ubiquitin protein ligase 2 (*WWP2*) and golgi glycoprotein 1 (*GLG1*) genes, from 94 pangenome assemblies. These DNA sequences were aligned to the reference genomes, and subsequently, we determined the protein sequences of CLEC18A, CLEC18B, and CLEC18C, each comprising 446 amino acids, based on their corresponding coordinates. Following the filtering of ambiguous and truncated CLEC18 protein sequences, we identified 90, 91, and 89 protein sequences for CLEC18A, CLEC18B, and CLEC18C, respectively. A comparison of these CLEC18 protein sequences revealed 29 SAVs within CLEC18 protein paralogs (as summarized in Table 1).

**Table 1:** Identification of paralogous sequence variants (PSVs) and polymorphic variants (PVs) of CLEC18 protein sequences by Human Pangenome Reference Consortium (HPRC) assemblies

**Position**	**Variation**			**Number of assemblies**												**Total frequency**			**Variant type**^**a**^
				CLEC18A			CLEC18B			CLEC18C			Total						
aa10	R	/	Q	90	/	0	90	/	1	89	/	0	269	/	1	0.996	/	0.004	PV
aa11	G	/	V	90	/	0	89	/	2	89	/	0	268	/	2	0.993	/	0.007	PV
aa14	L	/	P	88	/	2	91	/	0	89	/	0	268	/	2	0.993	/	0.007	PV
**aa24**	A	/	T	90	/	0	1	/	90	88	/	1	179	/	91	0.663	/	0.337	**PSV**
**aa91**	I	/	T	1	/	89	91	/	0	71	/	18	163	/	107	0.604	/	0.396	**PSV**
aa99	G	/	S	89	/	1	91	/	0	89	/	0	269	/	1	0.996	/	0.004	PV
aa100	L	/	P	83	/	7	91	/	0	55	/	34	229	/	41	0.848	/	0.152	PV
aa118	A	/	V	77	/	13	91	/	0	88	/	1	256	/	14	0.948	/	0.052	PV
aa148	T	/	S	90	/	0	91	/	0	55	/	34	236	/	34	0.874	/	0.126	PV
aa151	T	/	M	69	/	21	91	/	0	89	/	0	249	/	21	0.922	/	0.078	PV
aa152	Q	/	L	90	/	0	91	/	0	88	/	1	269	/	1	0.996	/	0.004	PV
aa165	R	/	Q	90	/	0	90	/	1	89	/	0	269	/	1	0.996	/	0.004	PV
aa168	C	/	W	90	/	0	91	/	0	88	/	1	269	/	1	0.996	/	0.004	PV
**aa173**	A	/	T	90	/	0	0	/	91	88	/	1	178	/	92	0.659	/	0.341	**PSV**
aa174	A	/	V	90	/	0	91	/	0	56	/	33	237	/	33	0.878	/	0.122	PV
**aa185**	G	/	R	5	/	85	91	/	0	80	/	9	176	/	94	0.652	/	0.348	**PSV**
**aa196**	I	/	V	1	/	89	91	/	0	45	/	44	137	/	133	0.507	/	0.493	**PSV**
aa275	E	/	D	90	/	0	91	/	0	68	/	21	249	/	21	0.922	/	0.078	PV
aa307	D	/	N	89	/	1	91	/	0	67	/	22	247	/	23	0.915	/	0.085	PV
aa312	M	/	T	90	/	0	90	/	1	89	/	0	269	/	1	0.996	/	0.004	PV
aa324	R	/	S	90	/	0	91	/	0	68	/	21	249	/	21	0.922	/	0.078	PV
aa332	G	/	R	90	/	0	91	/	0	86	/	3	267	/	3	0.989	/	0.011	PV
aa339	S	/	R	87	/	3	91	/	0	89	/	0	267	/	3	0.989	/	0.011	PV
aa352	R	/	C	85	/	5	90	/	1	89	/	0	264	/	6	0.978	/	0.022	PV
**aa360**	I	/	T	90	/	0	9	/	82	89	/	0	188	/	82	0.696	/	0.304	**PSV**
aa393	T	/	S	90	/	0	91	/	0	71	/	18	252	/	18	0.933	/	0.067	PV
**aa421**	D	/	N	80	/	10	91	/	0	35	/	54	206	/	64	0.763	/	0.237	**PSV**
aa437	E	/	K	90	/	0	91	/	0	88	/	1	269	/	1	0.996	/	0.004	PV
aa441	R	/	W	90	/	0	90	/	1	89	/	0	269	/	1	0.996	/	0.004	PV

^a^*PSV* Paralogous sequence variant, *PV* polymorphic variant. The variants with varying dominant amino acid sequences across CLEC18A, CLEC18B, and CLEC18C were identified as PSVs and highlighted in bold font

Out of these SAVs, seven (at positions 24, 91, 173, 185, 196, 360, and 421) were confirmed as PSVs, as they displayed varying dominant amino acids across CLEC18A, CLEC18B, and CLEC18C (see Table 1). The remaining 22 SAVs were classified as polymorphic variants (PVs), as they exhibited the same dominant amino acids across CLEC18 protein paralogs (refer to Table 1). Among these PSVs, only one (at position 360) was exclusively observed in the T2T-CHM13v2.0 reference assembly, while the others were detected in both the GRCh38.p14 and T2T-CHM13v2.0 assemblies (as shown in Fig. 2B and Table 1). It's noteworthy that only two PSVs (at positions 24 and 173) displayed identical patterns of amino acid changes across CLEC18 protein sequences in both reference assemblies and in the majority of pangenome assemblies (refer to Fig. 2B and Table 1). One PSV (at position 196) exhibited the same pattern in both the reference assemblies of GRCh38.p14 and T2T-CHM13v2.0, but not in most pangenome assemblies (as indicated in Fig. 2B and Table 1). The patterns of the other two PSVs (at positions 91 and 421) and one PSV (at position 185) based on the GRCh38.p14 assembly and the T2T-CHM13v2.0 assembly were consistent with the results from pangenome assemblies, respectively (as shown in Fig. 2B and Table 1).

**Fig. 4:**
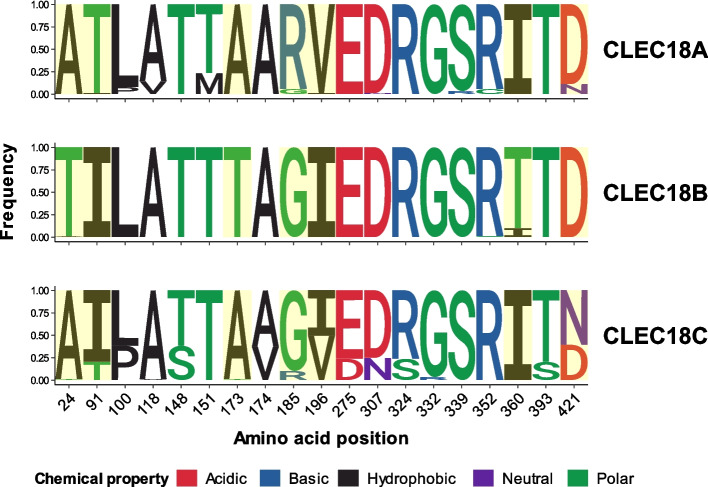
Diversity of paralogous sequence variants (PSVs) and polymorphic variants (PVs) of CLEC18 protein sequences in Human Pangenome Reference Consortium (HPRC) assemblies. Seven PSVs and 12 PVs were included after filtering PVs with an overall frequency of < 0.01 in three CLEC18 proteins. The height represents the percentage of amino acid changes of each PSV/PV. PSVs are highlighted in yellow

Furthermore, we investigated the diversity of amino acids at these PSV and PV positions within CLEC18 protein paralogs from pangenome assemblies and observed that the CLEC18C protein sequence exhibited more diversity, while the protein sequence of CLEC18B was notably more conserved than those of CLEC18A and CLEC18C (illustrated in Fig. 4). It is noteworthy that the amino acid changes of six PSVs (at positions 24, 91, 173, 185, 360, and 421) were also characterized by changes in chemical properties, including pH (acidic, basic, and neutral), hydrophobicity (hydrophobic), and polarity (polar). Among these PSVs, four of them (p.I91T, p.A173T, p.G185R, and p.I196V) are located on and near the CAP/SCP/TAPS domain (a.a.45-190), and two of them (p.I360T and p.D421N) are positioned at the CTLD (a.a.298-433) of CLEC18 protein paralogs. Additionally, we noted that eight of the common PVs (frequency > 0.01, at positions 148, 151, 174, 275, 324, 332, 339, and 393) were specific to either CLEC18A or CLEC18C in pangenome assemblies (refer to Table 1).

To corroborate the segmental duplication of the CLEC18 gene, we further validated the alignment of pangenome assemblies with the T2T-CHM13v2.0 reference assembly. Among the 94 pangenome assemblies, seven (HG00741-Pri-Mat, HG01109-Alt-Pat, HG01123-Alt-Pat, HG01361-Alt-Pat, HG01952-Alt-Pat, HG02148-Pri-Mat, and HG02559-Alt-Pat) successfully aligned to the CLEC18A locus in the T2T-CHM13v2.0 assembly without a deletion across the junction between CLEC18A duplicated segments (refer to Fig. S3). Furthermore, we performed sequencing alignment with 11 long-read whole-genome sequencing (WGS) datasets to the T2T-CHM13v2.0 reference genome. The long reads from four WGS datasets (HG004, HG00597, HG00609, and NA18747) were successfully aligned to the duplicated regions of the CLEC18A gene in the T2T-CHM13v2.0 reference assembly (as depicted in Fig. S4). These results substantiate the segmental duplication of the CLEC18A gene as a common structural variation within the human genome.

### Establishment of human CLEC18 paralog-specific signatures by variant combinations

Following the identification of PSVs and PVs through pangenome assemblies, our subsequent goal was to characterize the combinations of these variants within CLEC18 protein paralogs. We aimed to determine whether these variant combinations could effectively differentiate CLEC18A, CLEC18B, and CLEC18C protein sequences. Our analysis revealed that CLEC18 protein paralogs in the majority of pangenome assemblies were dominated by specific combinations of seven PSVs. However, the PSV combinations within CLEC18C exhibited greater diversity compared to CLEC18A and CLEC18B (refer to Table 2). For both CLEC18A and CLEC18B, the PSV combinations shared by multiple pangenome assemblies matched those identified in the previous study and in either the GRCh38.p14 or T2T-CHM13v2.0 reference assemblies (refer to Table 2). In contrast, the most common PSV combinations within CLEC18C, shared among the majority of pangenome assemblies, were not observed in the previous study or the reference genomes (refer to Table 2). Only the less common combinations of CLEC18C were also identified in the previous study and the reference assemblies (refer to Table 2).

**Table 2:** Identification of paralogous sequence variant (PSV) combinations of CLEC18 protein sequences by Human Pangenome Reference Consortium (HPRC) assemblies

**Gene**	**Amino acid position of PSVs**							**Number of assemblies**	**Number of CLEC18A-Dup**	**Annotation**^**a**^	**Repeat**^**b**^
	24	91	173	185	196	360	421				
CLEC18A	A	T	A	R	V	I	D	75	0	CLEC18A (GRCh38.p14) CLEC18A (JBC-2015)	
	A	T	A	R	V	I	N	9	7	CLEC18A (T2T-CHM13v2.0) CLEC18C (JBC-2015)	□
	A	T	A	G	V	I	D	4	0	CLEC18A (JBC-2015) CLEC18C (T2T-CHM13v2.0)	△
	A	I	A	R	V	I	D	1	0		
	A	T	A	G	I	I	N	1	0		
CLEC18B	T	I	T	G	I	T	D	81	0	CLEC18B (T2T-CHM13v2.0) CLEC18B (JBC-2015)	
	T	I	T	G	I	I	D	9	0	CLEC18B (GRCh38.p14) CLEC18B (JBC-2015)	
	A	I	T	G	I	T	D	1	0	CLEC18B (JBC-2015)	
CLEC18C	A	I	A	G	I	I	N	44	0		
	A	I	A	G	V	I	D	18	0		
	A	T	A	G	V	I	D	17	0	CLEC18C (T2T-CHM13v2.0) CLEC18A (JBC-2015)	△
	A	I	A	R	V	I	N	8	0	CLEC18C (GRCh38.p14) CLEC18C (JBC-2015)	
	A	T	A	R	V	I	N	1	0	CLEC18A (T2T-CHM13v2.0) CLEC18C (JBC-2015)	□
	T	I	T	G	I	I	N	1	0		

^a^PSV combinations matching amino acid combinations in reference assemblies (GRCh38.p14 or T2T-CHM13v2.0) or reported by Huang *et al.* 2015 (JBC-2015) [8] were annotated

^b^PSV combinations repeatedly observed in CLEC18A and CLEC18C were marked by given shapes. The triangle and square symbols each indicate two different PSV combinations that were identified as duplicated in CLE18A and CLEC18C

We observed that certain PSV combinations were repeatedly found in both CLEC18A and CLEC18C of pangenome assemblies (refer to Table 2). These consistent combinations indicated a high degree of similarity in PSVs between CLEC18A and CLEC18C protein sequences, making it challenging to distinguish between these CLEC18 paralogs. Therefore, we expanded our analysis to include 12 common PVs of CLEC18 protein sequences and profiled the combinations of PSVs and PVs in CLEC18 protein paralogs within pangenome assemblies. Our findings showed that most of the PSV plus PV combinations were unique to CLEC18 paralogs in pangenome assemblies. Only two rare combinations were repeatedly observed in both CLEC18A and CLEC18C (refer to Table 3). While major PSV plus PV combinations were still evident in CLEC18A, CLEC18B, and CLEC18C in most pangenome assemblies, the diversity of PSV plus PV combinations within CLEC18A was comparable to that within CLEC18C in pangenome assemblies. Moreover, the most frequently observed PSV plus PV combinations in CLEC18B in pangenome assemblies aligned with those determined in the previous study and in either the GRCh38.p14 or T2T-CHM13v2.0 assembly (refer to Table 3). In contrast, the most common PSV plus PV combinations within CLEC18A in pangenome assemblies were consistent with those from the previous study, and matches between combinations in reference genomes and pangenome assemblies were only observed for the less common combinations of both CLEC18A and CLEC18C (refer to Table 3).

**Table 3:** Identification of paralogous sequence variant (PSV) plus polymorphic variant (PV) combinations of CLEC18 protein sequences by Human Pangenome Reference Consortium (HPRC) assemblies

**Gene**	**Amino acid position of PSVs and PVs**^**a**^																			**Number of assemblies**	**Number of CLEC18A-Dup**	**Annotation**^**b**^	**Repeat**^**c**^
	24	91	100	118	148	151	173	174	185	196	275	307	324	332	339	352	360	393	421				
CLEC18A	A	T	L	A	T	**T**	A	A	R	V	E	D	R	G	**S**	R	I	T	D	43	0	CLEC18A (JBC-2015)	
	A	T	L	A	T	**M**	A	A	R	V	E	D	R	G	**S**	R	I	T	D	17	0	CLEC18A (JBC-2015)	
	A	T	L	V	T	**T**	A	A	R	V	E	D	R	G	**S**	R	I	T	D	12	0	CLEC18A (GRCh38.p14) CLEC18A (JBC-2015)	
	A	T	P	A	T	**T**	A	A	R	V	E	D	R	G	**S**	C	I	T	N	5	5	CLEC18A (T2T-CHM13v2.0) CLEC18C (JBC-2015)	
	A	T	L	A	T	**M**	A	A	R	V	E	D	R	G	**R**	R	I	T	D	3	0		
	A	T	L	A	T	**T**	A	A	G	V	E	D	R	G	**S**	R	I	T	D	3	0		
	A	T	P	A	T	**T**	A	A	R	V	E	D	R	G	**S**	R	I	T	N	2	2	CLEC18C (JBC-2015)	
	A	I	L	A	T	**T**	A	A	R	V	E	D	R	G	**S**	R	I	T	D	1	0		
	A	T	L	A	T	**M**	A	A	R	V	E	D	R	G	**S**	R	I	T	N	1	0	CLEC18C (JBC-2015)	
	A	T	L	A	T	**T**	A	A	G	I	E	D	R	G	**S**	R	I	T	N	1	0		
	A	T	L	A	T	**T**	A	A	G	V	E	N	R	G	**S**	R	I	T	D	1	0		△
	A	T	L	V	T	**T**	A	A	R	V	E	D	R	G	**S**	R	I	T	N	1	0		□
CLEC18B	T	I	L	A	T	T	T	A	G	I	E	D	R	G	S	R	T	T	D	80	0	CLEC18B (T2T-CHM13v2.0) CLEC18B (JBC-2015)	
	T	I	L	A	T	T	T	A	G	I	E	D	R	G	S	R	I	T	D	9	0	CLEC18B (GRCh38.p14) CLEC18B (JBC-2015)	
	A	I	L	A	T	T	T	A	G	I	E	D	R	G	S	R	T	T	D	1	0	CLEC18B (JBC-2015)	
	T	I	L	A	T	T	T	A	G	I	E	D	R	G	S	C	T	T	D	1	0	CLEC18B (JBC-2015)	
CLEC18C	A	I	L	A	**T**	T	A	**A**	G	I	**E**	D	**R**	**G**	S	R	I	**T**	N	41	0		
	A	I	P	A	**S**	T	A	**V**	G	V	**D**	N	**S**	**G**	S	R	I	**S**	D	17	0		
	A	T	P	A	**S**	T	A	**V**	G	V	**E**	D	**R**	**G**	S	R	I	**T**	D	13	0	CLEC18C (T2T-CHM13v2.0)	
	A	I	L	A	**T**	T	A	**A**	R	V	**E**	D	**R**	**G**	S	R	I	**T**	N	8	0	CLEC18C (GRCh38.p14) CLEC18C (JBC-2015)	
	A	I	L	A	**T**	T	A	**A**	G	I	**E**	D	**R**	**R**	S	R	I	**T**	N	3	0		
	A	T	P	A	**S**	T	A	**V**	G	V	**D**	N	**S**	**G**	S	R	I	**T**	D	3	0		
	A	I	P	A	**S**	T	A	**A**	G	V	**D**	N	**S**	**G**	S	R	I	**S**	D	1	0		
	A	T	L	A	**T**	T	A	**A**	G	V	**E**	N	**R**	**G**	S	R	I	**T**	D	1	0		△
	A	T	L	V	**T**	T	A	**A**	R	V	**E**	D	**R**	**G**	S	R	I	**T**	N	1	0		□
	T	I	L	A	**T**	T	T	**A**	G	I	**E**	D	**R**	**G**	S	R	I	**T**	N	1	0		

^a^Common PVs with frequencies > 0.01 in all CLEC18 paralogs were included. Amino acid changes of PVs that were observed in only one CLEC18 paralog were determined to be CLEC18 paralog-specific and highlighted in bold font

^b^PSV plus common PV combinations matching amino acid combinations in reference assemblies (GRCh38.p14 or T2T-CHM13v2.0) or reported by Huang *et al.* 2015 (JBC-2015) [8] were annotated

^c^PSV plus common PV combinations repeatedly observed in CLEC18A and CLEC18C were marked by given shapes. The triangle and square symbols each indicate two different PSV plus common PV combinations that were identified as duplicated in CLE18A and CLEC18C

Based on these CLEC18 variant combinations, we further profiled the haplotypes of CLEC18 SAV combinations for each pangenome assembly. Our results indicated that 43% (37 out of 86) of pangenome assemblies shared the same haplotype of PSV combinations within CLEC18A, CLEC18B, and CLEC18C protein sequences (refer to Table S1). Regarding PSV plus PV combinations, the haplotypes of combinations involving PSVs and PVs within CLEC18 protein paralogs were more diverse (refer to Table S2). Nevertheless, the top three haplotypes were observed in 45% (39 out of 86) of pangenome assemblies.

To validate these findings, we further analyzed the combinations of PSVs and PVs within CLEC18 protein sequences from 11 long-read WGS datasets, based on PacBio HiFi sequencing. We discovered that the majority of the long sequencing reads aligned to the CLEC18B locus in the GRCh38.p14 and T2T-CHM13v2.0 reference assemblies also matched the PSV plus PV combinations of CLEC18B protein, as identified through pangenome assembly analysis (refer to Table S3). In contrast, many long reads, which were predicted to originate from either CLEC18A or CLEC18C loci based on the inference of their PSV plus PV combinations, were ambiguously aligned to the CLEC18A and CLEC18C loci in the reference genome assemblies (refer to Table S3). Notably, all subjects were predicted to have a common PSV plus PV combination of CLEC18B, which was identified in pangenome assemblies. However, the combinations of CLEC18A and CLEC18C were more diverse in these subjects, consistent with the previous results from pangenome assemblies. It is also worth mentioning that all non-rare combinations, observed in more than one subject, were identified in pangenome assemblies (refer to Table S3).

To investigate whether these combinations of PSVs and PVs from pangenome assemblies are expressed in mRNA, we subsequently analyzed four long-read whole-transcriptome sequencing (WTS) datasets generated by PacBio Iso-Seq. Our validation demonstrated that all PSV plus PV combinations from long RNA-sequencing reads matched those from pangenome assemblies (refer to Table S4). The sequencing alignment and the pangenome-based classification of CLEC18 paralogs were consistent for CLEC18A and CLEC18B but not for CLEC18C (refer to Table S4).

### Evolutionary connection and protein domain divergence of CLEC18 across humans and non-human primates

To explore the evolutionary relationships among CLEC18 paralogs and orthologs spanning human and other species, we conducted a comparison of CLEC18/CLEC18-like protein sequences between humans and non-human primates through phylogenetic analysis. In this analysis, we included three representative CLEC18 proteins with 446 amino acids encoded by RefSeq/MANE select CLEC18 transcripts, which stood for CLEC18A, CLEC18B, and CLEC18C in humans. Additionally, we incorporated 31 consensus CLEC18/CLEC18-like protein sequences, identified as having 444-448 amino acids, from 25 non-human primates. The resulting phylogenetic tree, constructed based on amino acid sequences, revealed that the CLEC18A, CLEC18B, and CLEC18C proteins in humans (*Homo sapiens*) exhibited the closest affinities with CLEC18/CLEC18-like proteins found in chimpanzees (*Pan troglodytes*) and bonobos (*Pan paniscus*). Furthermore, these human CLEC18 proteins demonstrated close relationships with proteins from western lowland gorillas (*Gorilla gorilla*) (see Fig. S5). It's worth noting that these non-human primates also possess multiple CLEC18/CLEC18-like genes. Conversely, the sequences of human CLEC18 protein paralogs displayed lower degrees of similarity to CLEC18/CLEC18-like protein sequences from other non-human primates, especially those species with only a single CLEC18/CLEC18-like gene in their genomes (refer to Fig. S5).

**Fig. 5:**
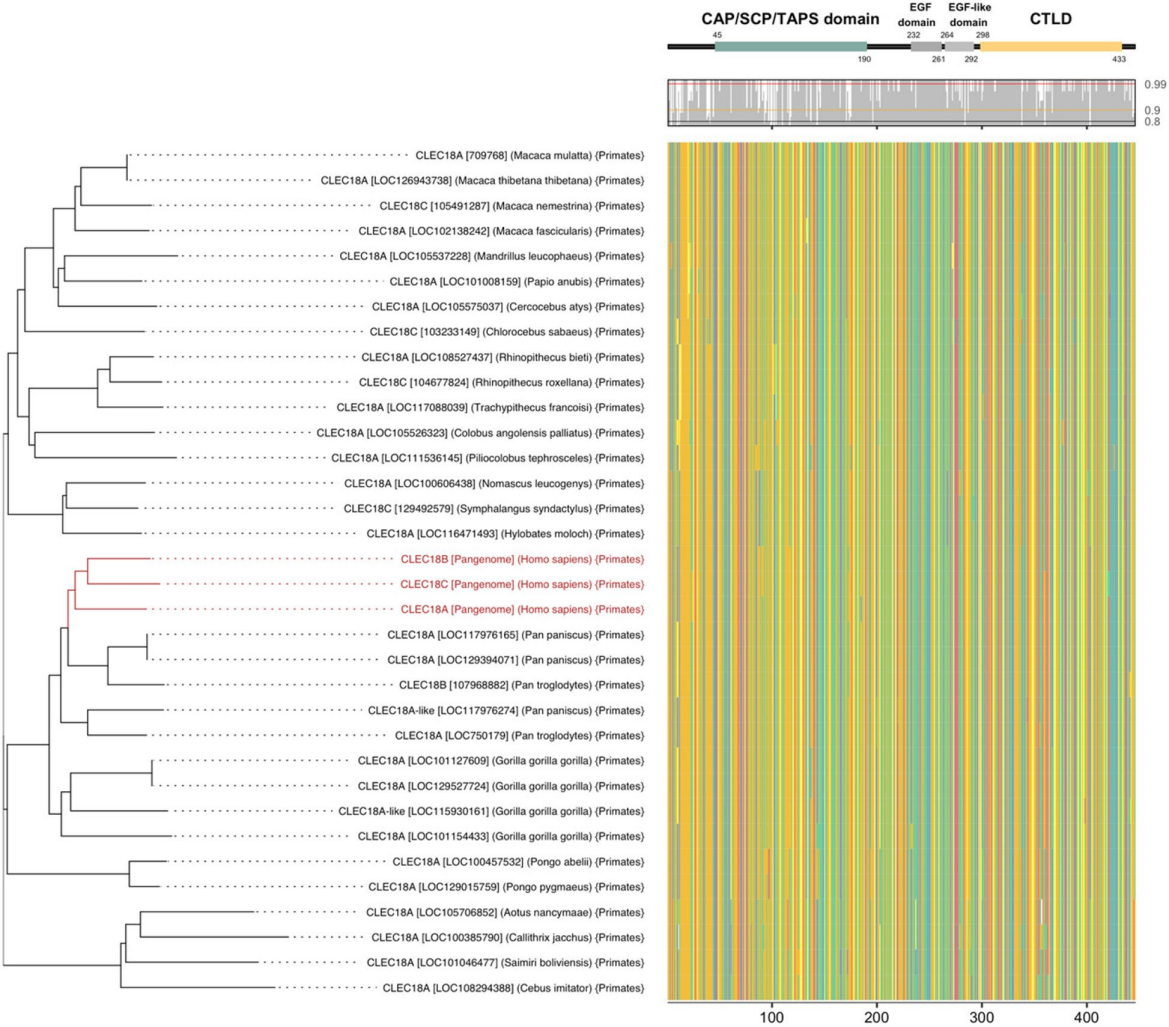
Comparison of CLEC18 protein sequences between humans and non-human primates. Multiple sequence alignment (MSA) of CLEC18 consensus protein sequences of humans and non-human primates is shown. For humans, population-based consensus CLEC18 protein sequences were determined by the human pangenome. As for non-human primates, predicted CLEC18 proteins of 444-448 amino acids of individual CLEC18 genes were used to determine consensus CLEC18 protein sequences. The phylogenetic tree was generated from MSAs of consensus CLEC18 protein sequences of humans and non-human primates. Sequence conservation and CLEC18 domains are shown above the MSA. Sequence conservation represents frequencies of major amino acids at individual positions across CLEC18 proteins of various species

Through the analysis of pangenome assemblies, we identified consensus sequences within human CLEC18 protein paralogs. These consensus CLEC18 protein sequences were considered as representatives of CLEC18A, CLEC18B, and CLEC18C instead of relying on reference CLEC18 paralogs within the human population. Consequently, we proceeded to conduct phylogenetic analysis by comparing population-based human CLEC18 protein sequences with those of non-human primates' CLEC18/CLEC18-like proteins (refer to Fig. 5). Our findings showed that the population-based human CLEC18A, CLEC18B, and CLEC18C protein sequences exhibited a closer relationship with each other in the phylogenetic tree. The close relationship between CLEC18/CLEC18-like protein sequences in humans, chimpanzees, and bonobos was still evident. These results were consistent with the phylogenetic analysis based on human reference CLEC18; however, the sequences of population-based human CLEC18 protein paralogs formed a tighter cluster and were more divergent from CLEC18/CLEC18-like protein sequences of our closest human relatives.

We also delved into the extent of divergence within CLEC18 protein sequences across humans and non-human primates. Our findings revealed that amino acid diversity was more pronounced in the CAP/SCP/TAPS and EGF-like domains compared to the EGF domain and CTLD across CLEC18/CLEC18-like protein sequences in both humans and non-human primates (see Figs. 5 and S5). While the EGF domain exhibited high conservation with over 90% similarity at every amino acid position, the CAP/SCP/TAPS domain displayed several highly variable positions with less than 90% similarities across various CLEC18/CLEC18-like protein sequences. By comparing pairwise dissimilarity of amino acid sequences across humans and non-human primates between CLEC18 domains, we demonstrated that the CAP/SCP/TAPS and EGF-like domains were more diverse than the EGF domain and CTLD (refer to Fig. S6). In summary, our study underscored that the N-terminal region of CLEC18 protein sequences showed greater divergence compared to the C-terminal region across humans and non-human primates (as depicted in Fig. 5).

### Investigation of unique amino acid alterations specific to human CLEC18 paralogs

**Fig. 6:**
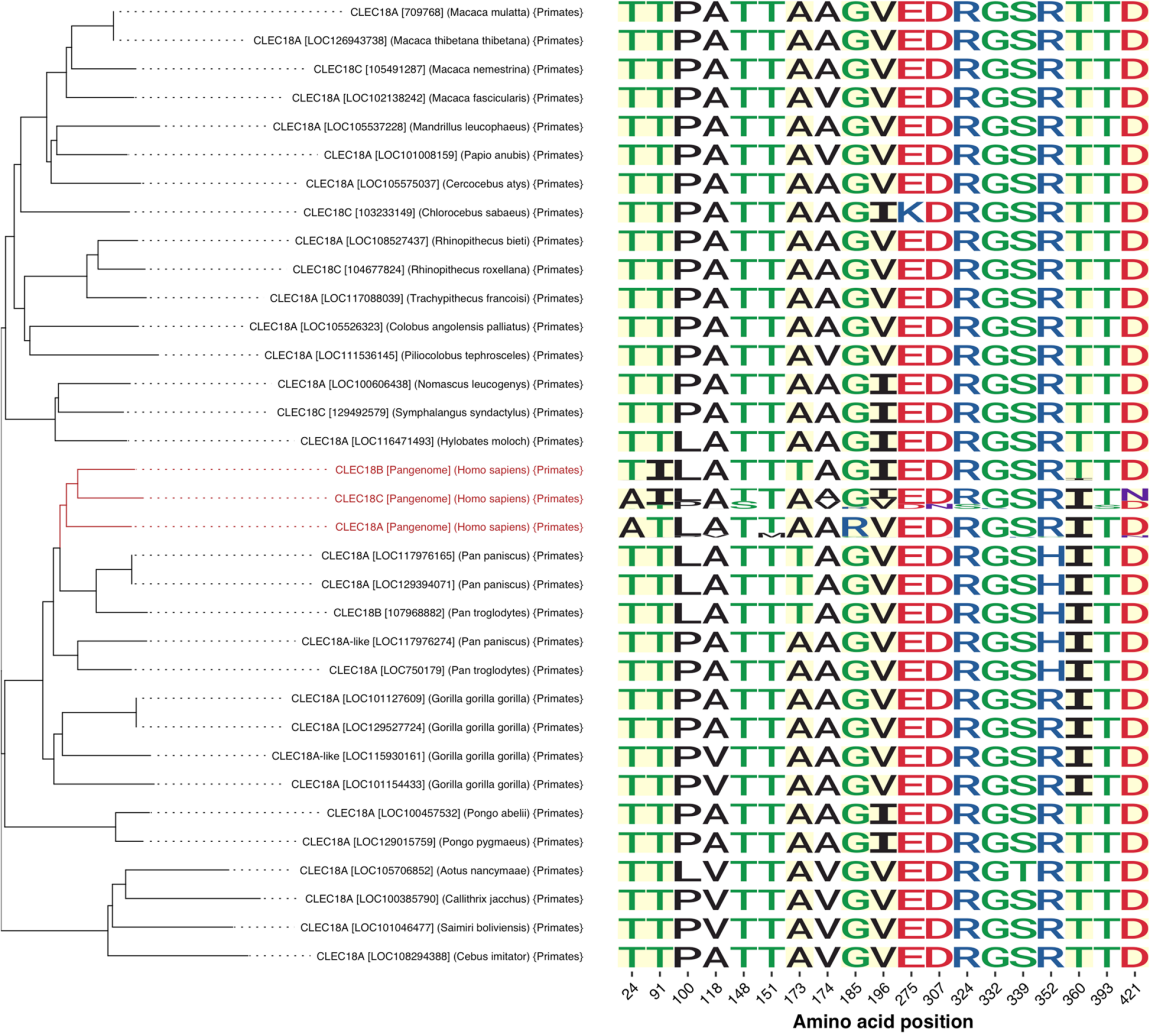
Comparison of amino acids at paralogous sequence variant (PSV) and polymorphic variant (PV) positions of CLEC18 protein sequences between humans and non-human primates. The phylogenetic tree was generated from multiple sequence alignments of consensus sequences of human pangenome CLEC18 protein paralogs and non-human primate reference CLEC18/CLEC18-like proteins. Amino acids of seven PSVs and 12 common PVs of humans and non-human primates were respectively based on CLEC18 protein sequences from human pangenome assemblies and NCBI non-primate reference genomes. The amino acid variation of human pangenome CLEC18 protein paralogs is shown by a logo plot. The positions of PSVs are highlighted in yellow

We further compared the amino acid sequences at PSV and PV positions of pangenome-derived human CLEC18A, CLEC18B, and CLEC18C with CLEC18/CLEC18-like of non-human primates. We observed variations in CLEC18/CLEC18-like protein sequences between humans and non-human primates (refer to Fig. 6). Specifically, amino acid changes at four PSV positions (Thr24 → Ala24, Thr91 → Ile91, Gly185 → Arg185, and Asp421 → Asn421) were specific to human CLEC18 paralogs. Among these changes, the alterations at a.a.185 (Gly185 → Arg185) and a.a.421 (Asp421 → Asn421) were predominantly identified in population-based human CLEC18A and CLEC18C, respectively. Additionally, the amino acid change at a.a.360 (Thr360 → Ile360) was detected in population-based human CLEC18A and CLEC18C, as well as in CLEC18/CLEC18-like protein sequences of other hominines, including chimpanzees, bonobos, and gorillas. Regarding common PVs, we found that four amino acid changes at a.a.148 (Thr148 → Ser148), a.a.151 (Thr151 → Met151), a.a.324 (Arg324 → Ser324), and a.a.393 (Thr393 → Ser393) had relatively higher frequencies and were only detected in either CLEC18C or CLEC18A protein sequences within human pangenome assemblies. In contrast, other PVs exhibited greater diversity across CLEC18/CLEC18-like protein sequences within human pangenomes and the reference genomes of non-human primates.

## Discussion

**Fig. 7:**
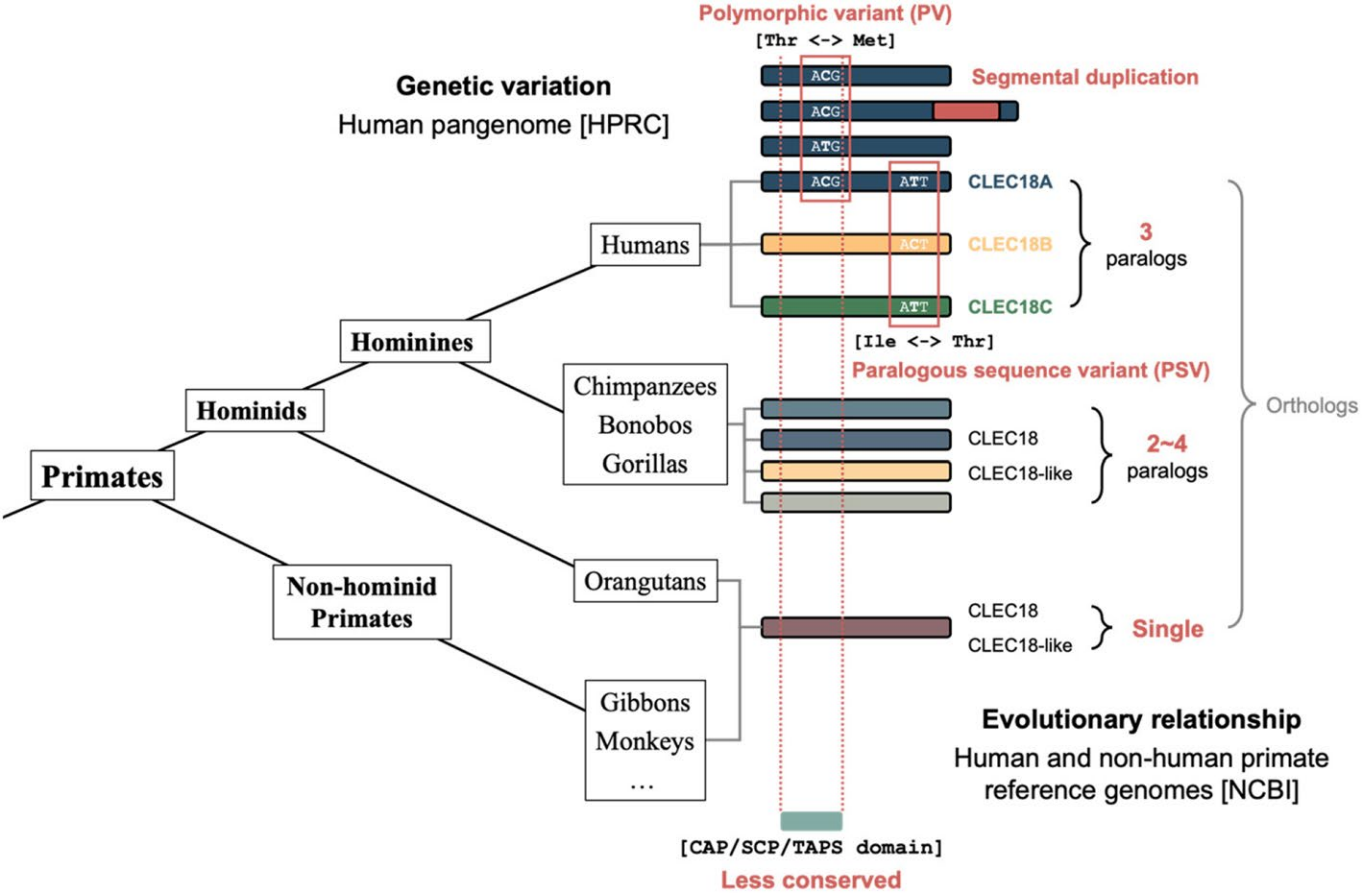
Summary of the study. The main findings include: (1) reliable paralogous sequence variants (PSVs) and polymorphic variants (PVs) of human CLEC18A, CLEC18B, and CLEC18C protein sequences; (2) A novel segmental duplication on human CLEC18A locus; (3) the evolutionary relationship in CLEC18 protein sequences and numbers between humans and non-human primates; (4) the less conservation of CAP/SCP/TAPS domain across CLEC18 protein sequences of humans and non-human primates

This study conducted a comprehensive exploration of the genomic and protein sequence variations within human CLEC18 paralogs. Furthermore, it delved into the homology of CLEC18 protein sequences in both humans and non-human primates. By leveraging long-read-based human reference genome and pangenome assemblies, a comprehensive analysis of paralogous sequence variants (PSVs) and polymorphic variants (PVs) within human CLEC18A, CLEC18B, and CLEC18C protein sequences was performed. One particularly intriguing discovery was the identification of a novel segmental duplication within the CLEC18A locus. Moreover, the study demonstrated the significance of combinations of amino acids at PSV and PV positions in distinguishing CLEC18 paralogous protein sequences through long-read DNA or RNA sequencing data. Through phylogenetic analysis, it was discovered that CLEC18A, CLEC18B, and CLEC18C proteins in humans (*Homo sapiens*) shared a closer relationship in sequence and number with CLEC18/CLEC18-like proteins found in chimpanzees (*Pan troglodytes*), bonobos (*Pan paniscus*), and gorillas (*Gorilla gorilla*). A multiple sequence alignment of CLEC18 protein sequences revealed higher diversity in amino acid sequences within the CAP/SCP/TAPS and EGF-like domains compared to the EGF domain and CTLD across CLEC18/CLEC18-like protein sequences in both humans and non-human primates. These findings provide comprehensive insights into the sequence divergence and evolutionary connection within human CLEC18 paralogs (see Fig. 7).

The primary challenge in studying human CLEC18 paralogs lies in their remarkable sequence similarity within CLEC18A, CLEC18B, and CLEC18C. Despite the substantial efforts of previous studies in demonstrating the significance of CLEC18 in innate immunity and its lipid-binding abilities [6–9], the difficulty in distinguishing between human CLEC18A, CLEC18B, and CLEC18C has limited our understanding of each specific CLEC18 paralog. Recent advancements in long-read sequencing technologies have led to more complete and accurate human genome assemblies [10, 13]. By harnessing long-read-based human pangenome assemblies, the study comprehensively profiled amino acid alterations between and within CLEC18A, CLEC18B, and CLEC18C protein sequences within the human population. Notably, some amino acid changes across CLEC18 protein paralogs observed in human reference genomes, including the GRCh38 and T2T-CHM13 assemblies, were attributed to polymorphic variations between individuals rather than paralogous sequence variations across paralogs. In other words, the false identification of PSVs for CLEC18 in reference genomes may arise due to PVs specific to certain CLEC18 paralogs. Consequently, utilizing human pangenome assemblies offers a more effective approach to uncovering CLEC18 paralog-specific signatures for distinguishing between human CLEC18A, CLEC18B, and CLEC18C. This approach led to the construction of signatures by composing combinations of amino acids at PSV and PV positions in CLEC18 protein sequences from pangenome assemblies. The strategy yielded highly distinct signatures for nearly all CLEC18 paralogs, with only two signatures showing overlap due to the high sequence resemblance between CLEC18A and CLEC18C. Subsequent assessments demonstrated that CLEC18 protein sequences from long-read-based whole-genome sequencing (WGS) and whole-transcriptome sequencing (WTS) could be accurately categorized into CLEC18 paralogs using these PSV and common PV signatures. Consequently, the study's results suggest a promising avenue for differentiating CLEC18 protein paralogs through human pangenome assemblies.

From the profiling of PSVs and PVs in human pangenome assemblies, several common PVs with paralog-specific characteristics were identified. For instance, the amino acid change p.T151M was exclusively found in human CLEC18A protein, supporting the idea that rs75776403, which leads to p.T151M and has been associated with phenotypic features in humans [9], represents a CLEC18A-specific genetic polymorphism. Other common CLEC18A- or CLEC18C-specific PVs, such as p.T148S, p.R324S, and p.T393S, may also be functionally significant, given their locations on either the CAP/SCP/TAPS domain or CTLD and their exclusive presence in human CLEC18 paralogs.

It's worth noting that a novel segmental duplication was detected within the CLEC18A locus in the human reference genome assembly of T2T-CHM13. Interestingly, this segmental duplication wasn't uncommon within the human population. Predictions suggested that the CDSs within the duplicated regions might generate distinct amino acid sequences for a portion of the CLEC18A protein. Consequently, individuals with this segmental duplication could potentially exhibit greater diversity in their CLEC18A mRNA and protein sequences, likely through alternative splicing across the duplicated sequences due to a *cis*-effect [19]. Exons on either the first, duplicated, or both segments might be utilized during CLEC18A transcription and translation, resulting in the production of diverse CLEC18A proteins. However, it's important to highlight that this hypothesis remains unverifiable at present due to the limited availability of datasets for long-read mRNA sequencing pertaining to human CLEC18A.

Based on the results of sequence homology, a further investigation of CLEC18/CLEC18-like protein sequences in humans and other closely related non-human primates (chimpanzees, bonobos, and gorillas) was conducted. It was observed that both humans and these closely related non-human primates possessed multiple paralogous CLEC18/CLEC18-like genes. In humans, specifically, there were three CLEC18 genes – CLEC18A, CLEC18B, and CLEC18C – all of which exhibited nearly identical sequences. This pattern was also found in chimpanzees, bonobos, and gorillas, which were considered the closest relatives of humans. The number of CLEC18 genes in these non-human primates was comparable to that in humans. However, in other non-human primates and non-primate mammals, such as carnivores, even-toed ungulates, and rodents, which are more phylogenetically distant from humans, only a single CLEC18 gene was identified. We thus surmised that the initial duplication event resulting in CLEC18 paralogous genes probably occurred in the ancestral lineage of hominines before the gorillini-hominini speciation. The emergence of CLEC18 paralogs likely facilitates functional divergence and coordination in the regulation of CLEC18 genes in humans and their closely related non-human primates [20, 21]. Given the crucial role of CLEC18 in innate immunity and lipid metabolism [6–9], CLEC18 paralogs in humans may have significant implications for immune diversity and metabolic regulation.

An observation from the study was that amino acid sequences within the CAP/SCP/TAPS domain appeared to be less conserved than those within the CTLD of CLEC18/CLEC18-like proteins across humans and non-human primates. This observation could potentially be attributed to divergent functions of these two domains. The CAP/SCP/TAPS domain has been implicated in binding to sterols, acidic glycolipids, and acidic phospholipids, all of which are related to lipid metabolism [22, 23]. In contrast, the CTLD is important for binding glycans and polysaccharides, playing a key role in innate immune responses [1]. Consequently, the higher sequence conservation of CTLD across CLEC18 proteins across species might be attributed to the need for recognizing common pathogen-associated molecular patterns (PAMPs) by the innate immune system [7, 24, 25]. In contrast, the higher sequence diversity in CAP/SCP/TAPS domains across CLEC18 protein sequences of humans and non-human primates could be due to evolutionary divergence in metabolic regulation of lipids. Additionally, the amino acid changes of PSVs with modified chemical properties in the CAP/SCP/TAPS domain or CTLD might have more pronounced effects on CLEC18 proteins. This could lead to diverse functionalities of CLEC18 and, consequently, varying binding capabilities of CLEC18 paralogs to different lipids and glycans. The diversity of CAP/SCP/TAPS domains and CTLD of CLEC18 paralogs may contribute to metabolic regulation and innate immunity.

## Conclusions

In summary, our research has identified paralogous and polymorphic variants within human CLEC18, enabling the differentiation of human CLEC18 protein paralogs. Furthermore, we have made an unprecedented discovery of a segmental duplication within CLEC18A. Moreover, our study has unveiled the evolutionary similarities in CLEC18 protein sequences between humans and non-human primates, suggesting an evolutionary history of CLEC18 paralogy and the evolutionary divergence between CLEC18 protein domains. These findings have enhanced our comprehension of human CLEC18 paralogs, opening the door to further investigations into their functional distinctions and varying expressions among CLEC18A, CLEC18B, and CLEC18C in humans. We anticipate that future studies will unveil more significant roles played by human CLEC18 paralogs in immune system functioning and metabolic regulation.

### Supplementary Information

Additional file 1: Table S1. Identification of paralogous sequence variant (PSV) combinations of CLEC18A-CLEC18B-CLEC18C protein sequences by Human Pangenome Reference Consortium (HPRC) datasets. Table S2. Identification of paralogous sequence variant (PSV) plus polymorphic variant (PV) combinations of CLEC18A-CLEC18B-CLEC18C protein sequences by Human Pangenome Reference Consortium (HPRC) datasets. Table S3. Validation of paralogous sequence variant (PSV) plus polymorphic variant (PV) combinations of CLEC18 protein sequences by human long-read whole-genome sequencing (WGS) data. Table S4. Validation of paralogous sequence variant (PSV) plus polymorphic variant (PV) combinations of CLEC18 protein sequences by human long-read whole-transcriptome sequencing (WTS) data. Figure S1. Identification of paralogous sequence variants (PSVs) of CLEC18 protein sequences in the GRCh38.p14 and T2T-CHM13v2.0 human reference genome assemblies. Figure S2. Comparison of *CLEC18A* gene sequence between the T2T-CHM13v2.0 and GRCh38.p14 reference assemblies. Figure S3. Mapping of Human Pangenome Reference Consortium (HPRC) human genome assemblies to the duplicated region of the *CLEC18A* gene in the T2T-CHM13v2.0 reference assembly. Figure S4. Mapping of Human Pangenome Reference Consortium (HPRC) human genome assemblies to the duplicated region of the *CLEC18A* gene in the T2T-CHM13v2.0 reference assembly. Figure S5. Comparison of reference CLEC18 protein sequences between humans and non-human primates. Figure S6. Comparison of sequence dissimilarities between CLEC18 protein domains.

## Data Availability

All the data used in this study are publicly accessible online. For instance, gene and protein sequences from both human and non-human primate reference genome assemblies can be obtained through the NCBI gene database (https://www.ncbi.nlm.nih.gov/gene). Human pangenome assemblies are accessible via the Ensembl project (https://projects.ensembl.org/hprc). If you're interested in PacBio long-read whole-genome sequencing (WGS) datasets like HG002, HG003, and HG004, you can find them at PacBio's official data repository (https://www.pacb.com/connect/datasets). Additionally, the WGS datasets for HG00408, HG00423, HG00544, HG00558, HG00597, HG00609, NA18612, and NA18747 are available through the NCBI Sequence Read Archive (SRA) database (PRJNA701308). If you require PacBio long-read whole-transcriptome sequencing (WTS) datasets, you can access those for human brain samples with Alzheimer's disease and universal human reference RNA (UHRR) from PacBio's data repository (https://www.pacb.com/connect/datasets). Furthermore, the WTS datasets for XpressRef Universal Total RNA (Qiagen(R)) and Human Brain Reference RNA (Ambion) spiked with 2% Lexogen SIRV-Set 4 can be found in the SRA database (PRJNA669886 and PRJNA777579).

## Abbreviations

CAP: Cysteine-rich secretory protein/antigen 5/pathogenesis-related 1
CDS: Coding region sequence
CLEC18: C-type lectin family 18
CRD: Carbohydrate recognition domain
CTLD: C-type lectin-like domain
DENV: Dengue virus
EGF: Epidermal growth factor
GLG1: Golgi glycoprotein 1
HBV: Hepatitis B virus
HCV: Hepatitis C virus
HiFi: High-fidelity
HPRC: Human Pangenome Reference Consortium
IAV: Influenza A virus
NCBI: National Center for Biotechnology Information
PAMP: Pathogen-associated molecular pattern
PSV: Paralogous sequence variant
PV: Polymorphic variant
SAV: Single amino acid variant
SCP: Sperm-coating glycoprotein
SRA: Sequence read archive
TAPS: Tpx 1/antigen 5/pathogenesis related-1/Sc7
WES: Whole-exome sequencing
WGS: Whole-genome sequencing
WTS: Whole-transcriptome sequencing
WWP2: WW domain containing E3 ubiquitin protein ligase 2
