# Supplementary material for: Characterization of the genetic variation and evolutionary divergence of the CLEC18 family
Source: J Biomed Sci. 2024 May 20;31:53. doi: 10.1186/s12929-024-01034-5 (PMC11103991; doi:10.1186/s12929-024-01034-5)
Supplement: Supplementary file 1 — Additional file 1: Table S1. Identification of paralogous sequence variant (PSV) combinations of CLEC18A-CLEC18B-CLEC18C protein sequences by Human Pangenome Reference Consortium (HPRC) datasets. Table S2. Identification of paralogous sequence variant (PSV) plus polymorphic variant (PV) combinations of CLEC18A-CLEC18B-CLEC18C protein sequences by Human Pangenome Reference Consortium (HPRC) datasets. Table S3. Validation of paralogous sequence variant (PSV) plus polymorphic variant (PV) combinations of CLEC18 protein sequences by human long-read whole-genome sequencing (WGS) data. Table S4. Validation of paralogous sequence variant (PSV) plus polymorphic variant (PV) combinations of CLEC18 protein sequences by human long-read whole-transcriptome sequencing (WTS) data. Figure S1. Identification of paralogous sequence variants (PSVs) of CLEC18 protein sequences in the GRCh38.p14 and T2T-CHM13v2.0 human reference genome assemblies. Figure S2. Comparison of CLEC18A gene sequence between the T2T-CHM13v2.0 and GRCh38.p14 reference assemblies. Figure S3. Mapping of Human Pangenome Reference Consortium (HPRC) human genome assemblies to the duplicated region of the CLEC18A gene in the T2T-CHM13v2.0 reference assembly. Figure S4. Mapping of Human Pangenome Reference Consortium (HPRC) human genome assemblies to the duplicated region of the CLEC18A gene in the T2T-CHM13v2.0 reference assembly. Figure S5. Comparison of reference CLEC18 protein sequences between humans and non-human primates. Figure S6. Comparison of sequence dissimilarities between CLEC18 protein domains. [file 12929_2024_1034_MOESM1_ESM.pdf]

**Table S1** Identification of paralogous sequence variant (PSV) combinations of CLEC18A-CLEC18B-CLEC18C protein sequences by Human Pangenome Reference Consortium (HPRC) datasets.

| **Amino acid position of PSVs** (24,91,173,185,196,360,421) | | | **Number of assemblies** | **Number of CLEC18A-Dup** |
|---|---|---|---|---|
| CLEC18A | CLEC18B | CLEC18C | | |
| A,T,A,R,V,I,D | T,I,T,G,I,T,D | A,I,A,G,I,I,N | 37 | 0 |
| A,T,A,R,V,I,D | T,I,T,G,I,T,D | A,I,A,G,V,I,D | 11 | 0 |
| A,T,A,R,V,I,D | T,I,T,G,I,T,D | A,T,A,G,V,I,D | 9 | 0 |
| A,T,A,R,V,I,D | T,I,T,G,I,T,D | A,I,A,R,V,I,N | 7 | 0 |
| A,T,A,R,V,I,N | T,I,T,G,I,T,D | A,T,A,G,V,I,D | 7 | 7 |
| A,T,A,R,V,I,D | T,I,T,G,I,I,D | A,I,A,G,I,I,N | 4 | 0 |
| A,T,A,G,V,I,D | T,I,T,G,I,T,D | A,I,A,G,V,I,D | 3 | 0 |
| A,I,A,R,V,I,D | T,I,T,G,I,T,D | A,I,A,R,V,I,N | 1 | 0 |
| A,T,A,G,I,I,N | T,I,T,G,I,T,D | A,I,A,G,V,I,D | 1 | 0 |
| A,T,A,G,V,I,D | T,I,T,G,I,I,D | A,I,A,G,V,I,D | 1 | 0 |
| A,T,A,R,V,I,D | A,I,T,G,I,T,D | A,I,A,G,I,I,N | 1 | 0 |
| A,T,A,R,V,I,D | T,I,T,G,I,I,D | A,T,A,G,V,I,D | 1 | 0 |
| A,T,A,R,V,I,D | T,I,T,G,I,I,D | T,I,T,G,I,I,N | 1 | 0 |
| A,T,A,R,V,I,N | T,I,T,G,I,I,D | A,I,A,G,I,I,N | 1 | 0 |
| A,T,A,R,V,I,N | T,I,T,G,I,T,D | A,T,A,R,V,I,N | 1 | 0 |

**Table S2** Identification of paralogous sequence variant (PSV) plus polymorphic variant (PV) combinations of CLEC18A-CLEC18B-CLEC18C protein sequences by Human Pangenome Reference Consortium (HPRC) datasets.

| **Amino acid position of PSVs and PVs** (24,91,100,118,148,151,173,174,185,196,275,307,324,332,339,352,360,393,421)[a] | | | **Number of assemblies** | **Number of CLEC18A-Dup** |
|---|---|---|---|---|
| CLEC18A | CLEC18B | CLEC18C | | |
| A,T,L,A,T,T,A,A,R,V,E,D,R,G,S,R,I,T,D | T,I,L,A,T,T,T,A,G,I,E,D,R,G,S,R,T,T,D | A,I,L,A,T,T,A,A,G,I,E,D,R,G,S,R,I,T,N | 17 | 0 |
| A,T,L,A,T,M,A,A,R,V,E,D,R,G,S,R,I,T,D | T,I,L,A,T,T,T,A,G,I,E,D,R,G,S,R,T,T,D | A,I,L,A,T,T,A,A,G,I,E,D,R,G,S,R,I,T,N | 11 | 0 |
| A,T,L,A,T,T,A,A,R,V,E,D,R,G,S,R,I,T,D | T,I,L,A,T,T,T,A,G,I,E,D,R,G,S,R,T,T,D | A,I,P,A,S,T,A,V,G,V,D,N,S,G,S,R,I,S,D | 11 | 0 |
| A,T,L,V,T,T,A,A,R,V,E,D,R,G,S,R,I,T,D | T,I,L,A,T,T,T,A,G,I,E,D,R,G,S,R,T,T,D | A,I,L,A,T,T,A,A,R,V,E,D,R,G,S,R,I,T,N | 7 | 0 |
| A,T,L,A,T,T,A,A,R,V,E,D,R,G,S,R,I,T,D | T,I,L,A,T,T,T,A,G,I,E,D,R,G,S,R,T,T,D | A,T,P,A,S,T,A,V,G,V,E,D,R,G,S,R,I,T,D | 4 | 0 |
| A,T,P,A,T,T,A,A,R,V,E,D,R,G,S,C,I,T,N | T,I,L,A,T,T,T,A,G,I,E,D,R,G,S,R,T,T,D | A,T,P,A,S,T,A,V,G,V,E,D,R,G,S,R,I,T,D | 4 | 4 |
| A,T,L,A,T,M,A,A,R,V,E,D,R,G,R,R,I,T,D | T,I,L,A,T,T,T,A,G,I,E,D,R,G,S,R,T,T,D | A,I,L,A,T,T,A,A,G,I,E,D,R,G,S,R,I,T,N | 3 | 0 |
| A,T,L,A,T,M,A,A,R,V,E,D,R,G,S,R,I,T,D | T,I,L,A,T,T,T,A,G,I,E,D,R,G,S,R,T,T,D | A,I,L,A,T,T,A,A,G,I,E,D,R,R,S,R,I,T,N | 3 | 0 |
| A,T,L,A,T,T,A,A,R,V,E,D,R,G,S,R,I,T,D | T,I,L,A,T,T,T,A,G,I,E,D,R,G,S,R,I,T,D | A,I,L,A,T,T,A,A,G,I,E,D,R,G,S,R,I,T,N | 3 | 0 |
| A,T,L,A,T,T,A,A,R,V,E,D,R,G,S,R,I,T,D | T,I,L,A,T,T,T,A,G,I,E,D,R,G,S,R,T,T,D | A,T,P,A,S,T,A,V,G,V,D,N,S,G,S,R,I,T,D | 3 | 0 |
| A,T,L,V,T,T,A,A,R,V,E,D,R,G,S,R,I,T,D | T,I,L,A,T,T,T,A,G,I,E,D,R,G,S,R,T,T,D | A,I,L,A,T,T,A,A,G,I,E,D,R,G,S,R,I,T,N | 3 | 0 |
| A,T,L,A,T,T,A,A,G,V,E,D,R,G,S,R,I,T,D | T,I,L,A,T,T,T,A,G,I,E,D,R,G,S,R,T,T,D | A,I,P,A,S,T,A,V,G,V,D,N,S,G,S,R,I,S,D | 2 | 0 |
| A,T,P,A,T,T,A,A,R,V,E,D,R,G,S,R,I,T,N | T,I,L,A,T,T,T,A,G,I,E,D,R,G,S,R,T,T,D | A,T,P,A,S,T,A,V,G,V,E,D,R,G,S,R,I,T,D | 2 | 2 |
| A,I,L,A,T,T,A,A,R,V,E,D,R,G,S,R,I,T,D | T,I,L,A,T,T,T,A,G,I,E,D,R,G,S,R,T,T,D | A,I,L,A,T,T,A,A,R,V,E,D,R,G,S,R,I,T,N | 1 | 0 |
| A,T,L,A,T,M,A,A,R,V,E,D,R,G,S,R,I,T,D | A,I,L,A,T,T,T,A,G,I,E,D,R,G,S,R,T,T,D | A,I,L,A,T,T,A,A,G,I,E,D,R,G,S,R,I,T,N | 1 | 0 |
| A,T,L,A,T,M,A,A,R,V,E,D,R,G,S,R,I,T,D | T,I,L,A,T,T,T,A,G,I,E,D,R,G,S,R,T,T,D | A,T,L,A,T,T,A,A,G,V,E,N,R,G,S,R,I,T,D | 1 | 0 |
| A,T,L,A,T,M,A,A,R,V,E,D,R,G,S,R,I,T,D | T,I,L,A,T,T,T,A,G,I,E,D,R,G,S,R,T,T,D | A,T,P,A,S,T,A,V,G,V,E,D,R,G,S,R,I,T,D | 1 | 0 |
| A,T,L,A,T,M,A,A,R,V,E,D,R,G,S,R,I,T,N | T,I,L,A,T,T,T,A,G,I,E,D,R,G,S,R,I,T,D | A,I,L,A,T,T,A,A,G,I,E,D,R,G,S,R,I,T,N | 1 | 0 |
| A,T,L,A,T,T,A,A,G,I,E,D,R,G,S,R,I,T,N | T,I,L,A,T,T,T,A,G,I,E,D,R,G,S,R,T,T,D | A,I,P,A,S,T,A,V,G,V,D,N,S,G,S,R,I,S,D | 1 | 0 |
| A,T,L,A,T,T,A,A,G,V,E,D,R,G,S,R,I,T,D | T,I,L,A,T,T,T,A,G,I,E,D,R,G,S,R,I,T,D | A,I,P,A,S,T,A,V,G,V,D,N,S,G,S,R,I,S,D | 1 | 0 |
| A,T,L,A,T,T,A,A,G,V,E,N,R,G,S,R,I,T,D | T,I,L,A,T,T,T,A,G,I,E,D,R,G,S,R,T,T,D | A,I,P,A,S,T,A,A,G,V,D,N,S,G,S,R,I,S,D | 1 | 0 |
| A,T,L,A,T,T,A,A,R,V,E,D,R,G,S,R,I,T,D | T,I,L,A,T,T,T,A,G,I,E,D,R,G,S,R,I,T,D | A,T,P,A,S,T,A,V,G,V,E,D,R,G,S,R,I,T,D | 1 | 0 |
| A,T,L,A,T,T,A,A,R,V,E,D,R,G,S,R,I,T,D | T,I,L,A,T,T,T,A,G,I,E,D,R,G,S,R,I,T,D | T,I,L,A,T,T,T,A,G,I,E,D,R,G,S,R,I,T,N | 1 | 0 |
| A,T,L,V,T,T,A,A,R,V,E,D,R,G,S,R,I,T,D | T,I,L,A,T,T,T,A,G,I,E,D,R,G,S,R,I,T,D | A,I,L,A,T,T,A,A,G,I,E,D,R,G,S,R,I,T,N | 1 | 0 |

| | | | | |
|---|---|---|---|---|
| A,T,L,V,T,T,A,A,R,V,E,D,R,G,S,R,I,T,N | T,I,L,A,T,T,T,A,G,I,E,D,R,G,S,R,T,T,D | A,T,L,V,T,T,A,A,R,V,E,D,R,G,S,R,I,T,N | 1 | 0 |
| A,T,P,A,T,T,A,A,R,V,E,D,R,G,S,C,I,T,N | T,I,L,A,T,T,T,A,G,I,E,D,R,G,S,C,T,T,D | A,T,P,A,S,T,A,V,G,V,E,D,R,G,S,R,I,T,D | 1 | 1 |

[a] Common PVs with frequencies > 0.01 in all CLEC18 paralogs were included.

**Table S3** Validation of paralogous sequence variant (PSV) plus polymorphic variant (PV) combinations of CLEC18 protein sequences by human long-read whole-genome sequencing (WGS) data.

| Gene | Amino acid position of PSVs and PVs[a] | | | | | | | | | | | | | | | | | | | Number of samples | Number and gene of alignments | Annotation[b] |
|---|---|---|---|---|---|---|---|---|---|---|---|---|---|---|---|---|---|---|---|---|---|---|
| | 24 | 91 | 100 | 118 | 148 | 151 | 173 | 174 | 185 | 196 | 275 | 307 | 324 | 332 | 339 | 352 | 360 | 393 | 421 | | | |
| | A | T | L | A | T | **T** | A | A | R | V | E | D | R | G | **S** | R | I | T | D | 6 | CLEC18A (19)<br>CLEC18A&C (51) | CLEC18A (HPRC)<br>CLEC18A (JBC-2015) |
| | A | T | L | A | T | **M** | A | A | R | V | E | D | R | G | **S** | R | I | T | D | 5 | CLEC18A (10)<br>CLEC18A&C (22) | CLEC18A (HPRC)<br>CLEC18A (JBC-2015) |
| | A | T | P | A | T | **T** | A | A | R | V | E | D | R | G | **S** | C | I | T | N | 3 | CLEC18A (20) | CLEC18A (HPRC)<br>CLEC18A (T2T-CHM13v2.0)<br>CLEC18C (JBC-2015) |
| CLEC18A | A | I | L | A | T | **T** | A | A | R | V | E | D | R | G | **S** | R | I | T | D | 2 | CLEC18A (2)<br>CLEC18A&C (6)<br>CLEC18C (6) | CLEC18A (HPRC) |
| | A | T | L | A | T | **M** | A | A | R | V | E | D | R | G | **R** | R | I | T | D | 1 | CLEC18A (4)<br>CLEC18A&C (8) | CLEC18A (HPRC) |
| | A | T | L | V | T | **T** | A | A | R | V | E | D | R | G | **S** | R | I | T | D | 2 | CLEC18A (3)<br>CLEC18A&C (6) | CLEC18A (HPRC)<br>CLEC18A (GRCh38.p14)<br>CLEC18A (JBC-2015) |
| | A | T | L | A | T | **M** | A | A | R | V | E | D | R | G | **S** | G | I | T | D | 1 | CLEC18A (1) | CLEC18A (JBC-2015) |
| | A | T | P | A | T | **M** | A | A | R | V | E | D | R | G | **R** | R | I | T | D | 1 | CLEC18A (1) | |
| CLEC18B | T | I | L | A | T | T | T | A | G | I | E | D | R | G | S | R | T | T | D | 11 | CLEC18B (154)<br>CLEC18B&C (2)<br>CLEC18C (1) | CLEC18B (HPRC)<br>CLEC18B (T2T-CHM13v2.0)<br>CLEC18B (JBC-2015) |
| | T | I | L | A | T | T | T | A | G | I | E | D | R | G | S | R | I | T | D | 4 | CLEC18B (19) | CLEC18B (HPRC)<br>CLEC18B (GRCh38.p14) |

| | | | | | | | | | | | | | | | | | | | | | | |
|---|---|---|---|---|---|---|---|---|---|---|---|---|---|---|---|---|---|---|---|---|---|---|
| | | | | | | | | | | | | | | | | | | | | | | CLEC18B (JBC-2015) |
| | T | I | L | A | N | T | T | A | G | I | E | D | R | G | S | R | T | T | D | 1 | CLEC18B (1) | CLEC18B (JBC-2015) |
| | T | I | L | A | T | T | T | A | G | I | R | D | R | G | S | R | T | T | D | 1 | CLEC18B&C (1) | CLEC18B (JBC-2015) |
| CLEC18C | A | T | P | A | **S** | T | A | **V** | G | V | E | D | R | **G** | S | R | I | T | D | 6 | CLEC18A&C (27)<br>CLEC18C (20) | CLEC18C (HPRC)<br>CLEC18C (T2T-CHM13v2.0) |
| | A | I | L | A | **T** | T | A | **A** | R | V | E | D | R | **G** | S | R | I | T | N | 4 | CLEC18A&C (4)<br>CLEC18C (36) | CLEC18C (HPRC)<br>CLEC18C (GRCh38.p14)<br>CLEC18C (JBC-2015) |
| | A | I | L | A | **T** | T | A | **A** | G | I | E | D | R | **G** | S | R | I | T | N | 3 | CLEC18A&C (2)<br>CLEC18B&C (8)<br>CLEC18C (29) | CLEC18C (HPRC) |
| | T | I | L | A | **T** | T | T | **A** | G | I | E | D | R | **G** | S | R | I | T | N | 3 | CLEC18A&C (1)<br>CLEC18B (7)<br>CLEC18B&C (12)<br>CLEC18C (5) | CLEC18C (HPRC) |
| | A | I | L | A | **T** | T | A | **A** | G | I | E | D | R | **R** | S | R | I | T | N | 1 | CLEC18C (1) | CLEC18C (HPRC) |
| | A | I | L | A | **T** | T | A | **A** | R | I | E | D | R | **G** | S | R | I | T | N | 1 | CLEC18B&C (2)<br>CLEC18C (6) | |
| | A | I | P | A | **T** | T | A | **A** | R | I | E | D | R | **G** | S | R | I | T | N | 1 | CLEC18C (1) | |
| | A | T | P | A | **T** | T | A | **V** | G | V | E | D | R | **G** | S | R | I | T | D | 1 | CLEC18C (1) | |

[a] Common PVs with frequencies > 0.01 in all CLEC18 paralogs based on the analysis of pangenome assemblies were included. Amino acid changes of CLEC18 paralog-specific PVs observed in both pangenome and long-read WGS analyses were highlighted with bold font.

[b] PSV combinations matching amino acid combinations in reference assemblies (GRCh38.p14 or T2T-CHM13v2.0) or reported by Huang *et al.*, 2015 (JBC-2015) [1] were annotated.

**Table S4** Validation of paralogous sequence variant (PSV) plus polymorphic variant (PV) combinations of CLEC18 protein sequences by human long-read whole-transcriptome sequencing (WTS) data.

| Gene | Amino acid position of PSVs and PVs[a] | | | | | | | | | | | | | | | | | | | Number of samples | Number and gene of alignments | Annotation[b] |
|---|---|---|---|---|---|---|---|---|---|---|---|---|---|---|---|---|---|---|---|---|---|---|
| | 24 | 91 | 100 | 118 | 148 | 151 | 173 | 174 | 185 | 196 | 275 | 307 | 324 | 332 | 339 | 352 | 360 | 393 | 421 | | | |
| CLEC18A | A | T | L | A | T | **M** | A | A | R | V | E | D | R | G | S | R | I | T | D | 3 | CLEC18A (6) | CLEC18A (HPRC)<br>CLEC18A (JBC-2015) |
| | A | T | P | A | T | **T** | A | A | R | V | E | D | R | G | S | R | I | T | N | 1 | CLEC18A (2) | CLEC18A (HPRC)<br>CLEC18C (JBC-2015) |
| CLEC18B | T | I | L | A | T | T | T | A | G | I | E | D | R | G | S | R | T | T | D | 3 | CLEC18B (9) | CLEC18B (HPRC)<br>CLEC18B (JBC-2015)<br>CLEC18B (T2T-CHM13v2.0) |
| | T | I | L | A | T | T | T | A | G | I | E | D | R | G | S | R | I | T | D | 1 | CLEC18B (3) | CLEC18B (HPRC)<br>CLEC18B (GRCh38.p14)<br>CLEC18B (JBC-2015) |
| CLEC18C | A | T | P | A | **S** | T | A | **V** | G | V | E | D | R | G | S | R | I | T | D | 1 | CLEC18A (1) | CLEC18C (HPRC)<br>CLEC18C (T2T-CHM13v2.0) |

[a] Common PVs with frequencies > 0.01 in all CLEC18 paralogs based on the analysis of pangenome assemblies were included. Amino acid changes of CLEC18 paralog-specific PVs observed in both pangenome and long-read WTS analyses were highlighted with bold font.

[b] PSV combinations matching amino acid combinations in reference assemblies (GRCh38.p14 or T2T-CHM13v2.0) or reported by Huang *et al.*, 2015 (JBC-2015) [1] were annotated.

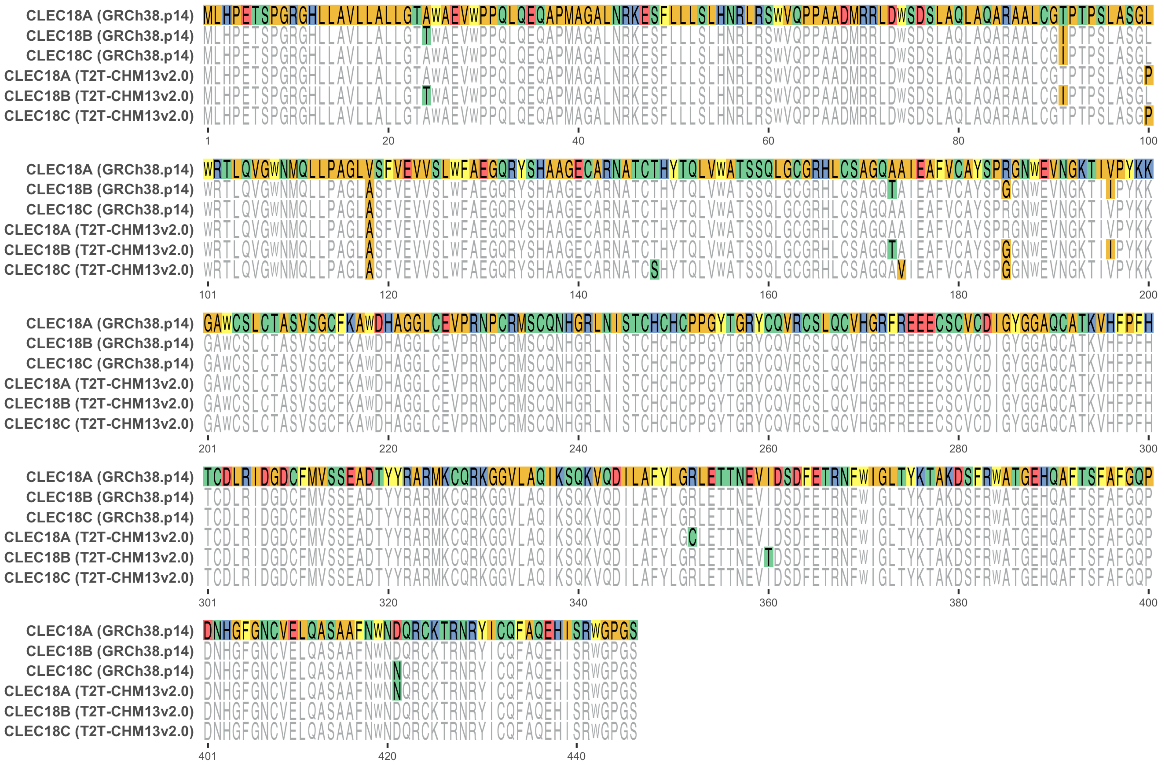

**Figure S1** Identification of paralogous sequence variants (PSVs) of CLEC18 protein sequences in the GRCh38.p14 and T2T-CHM13v2.0 human reference genome assemblies. Comparison of human CLEC18A, CLEC18B, and CLEC18C protein sequences from the GRCh38.p14 and T2T-CHM13v2.0 assemblies by multiple sequence alignments is shown.

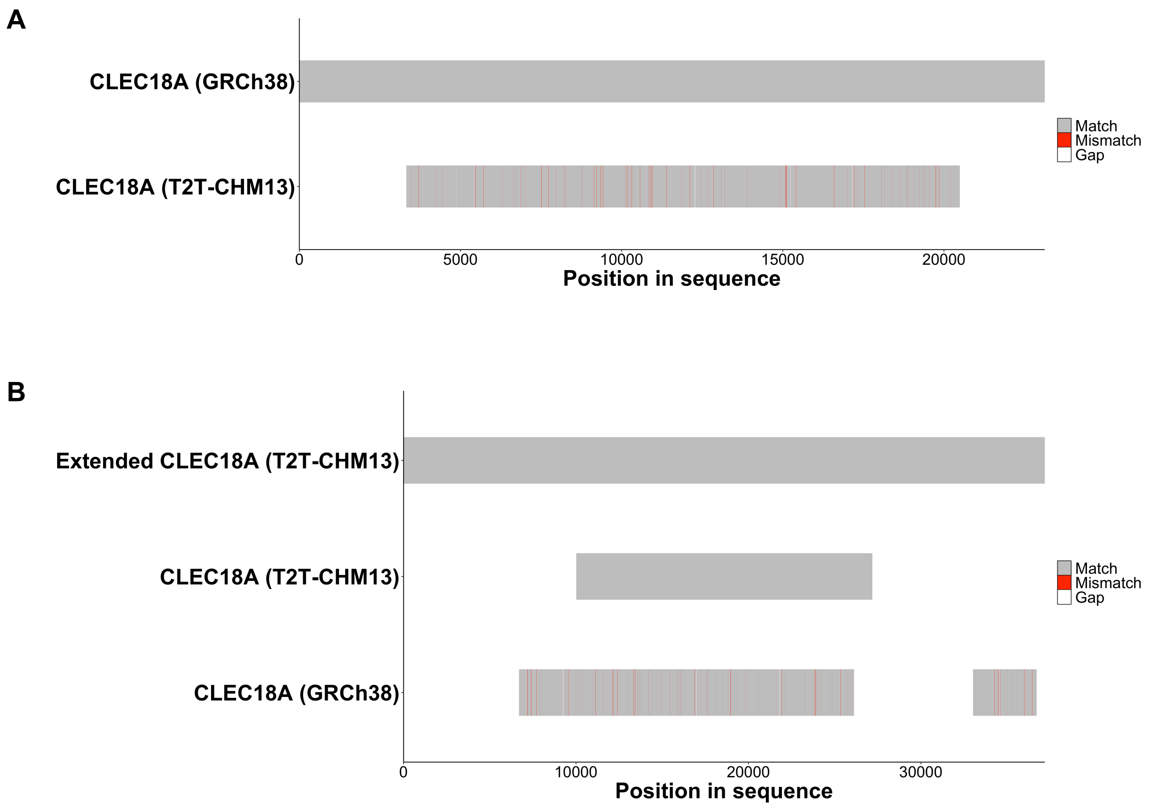


**Figure S2** Comparison of *CLEC18A* gene sequence between the T2T-CHM13v2.0 and GRCh38.p14 reference assemblies. (**A**) DNA sequences of the *CLEC18A* gene from the GRCh38.p14 and T2T-CHM13v2.0 reference assemblies were compared by multiple sequence alignments. (**B**) Comparison of DNA sequences between extended *CLEC18A* sequence (extension of 10,000 bp at both 3' and 5' ends) in the T2T-CHM13v2.0 and the *CLEC18A* gene in T2T-CHM13v2.0 and the *CLEC18A* gene in GRCh38.p14 was performed by a multiple sequence alignment.

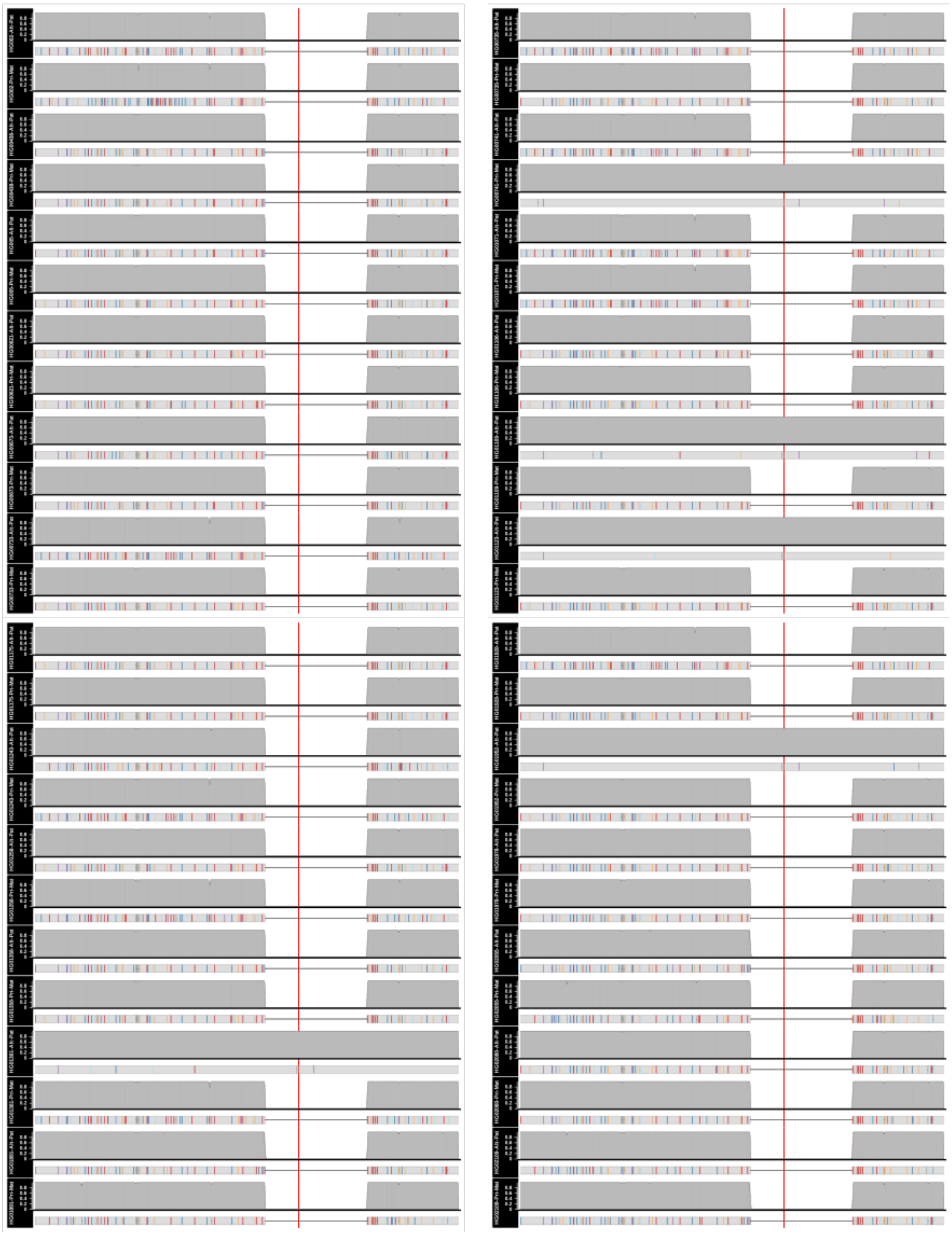

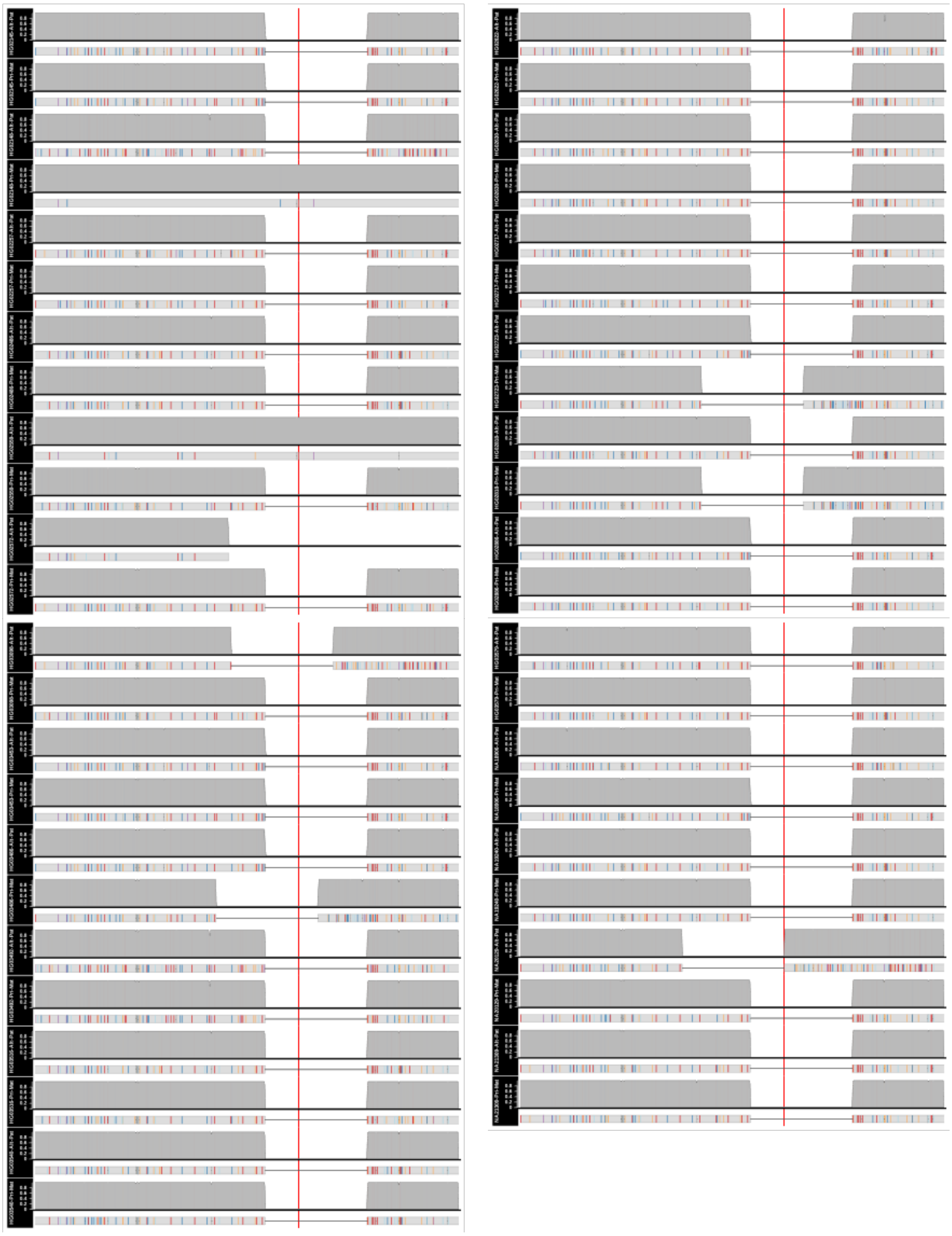

**Figure S3** Mapping of Human Pangenome Reference Consortium (HPRC) human genome assemblies to the duplicated region of the *CLEC18A* gene in the T2T-CHM13v2.0 reference assembly. 94 assemblies from 47 HPRC subjects were included.

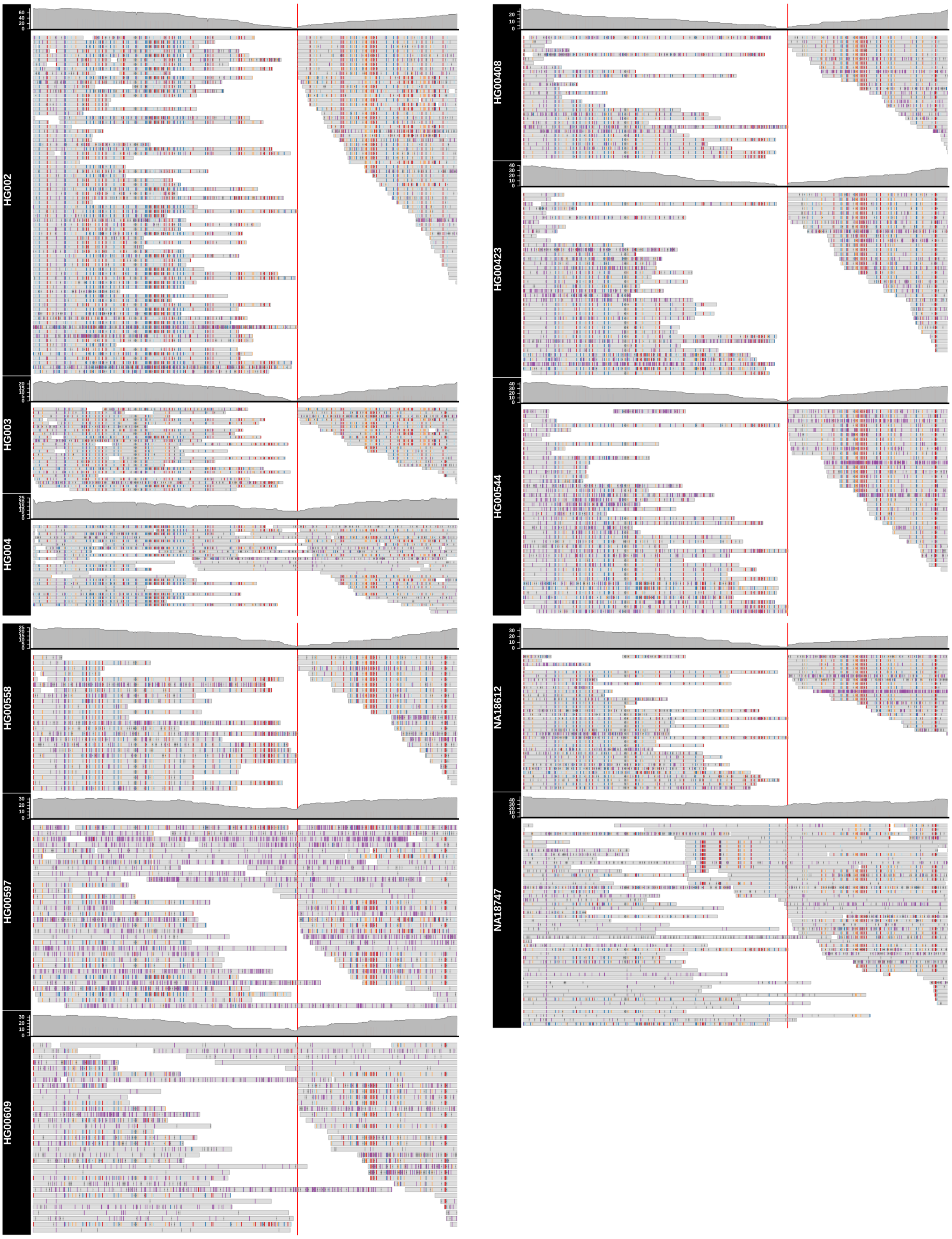


**Figure S4** Mapping of Human Pangenome Reference Consortium (HPRC) human genome assemblies to the duplicated region of the *CLEC18A* gene in the T2T-CHM13v2.0 reference assembly. 94 assemblies from 47 HPRC subjects were included.

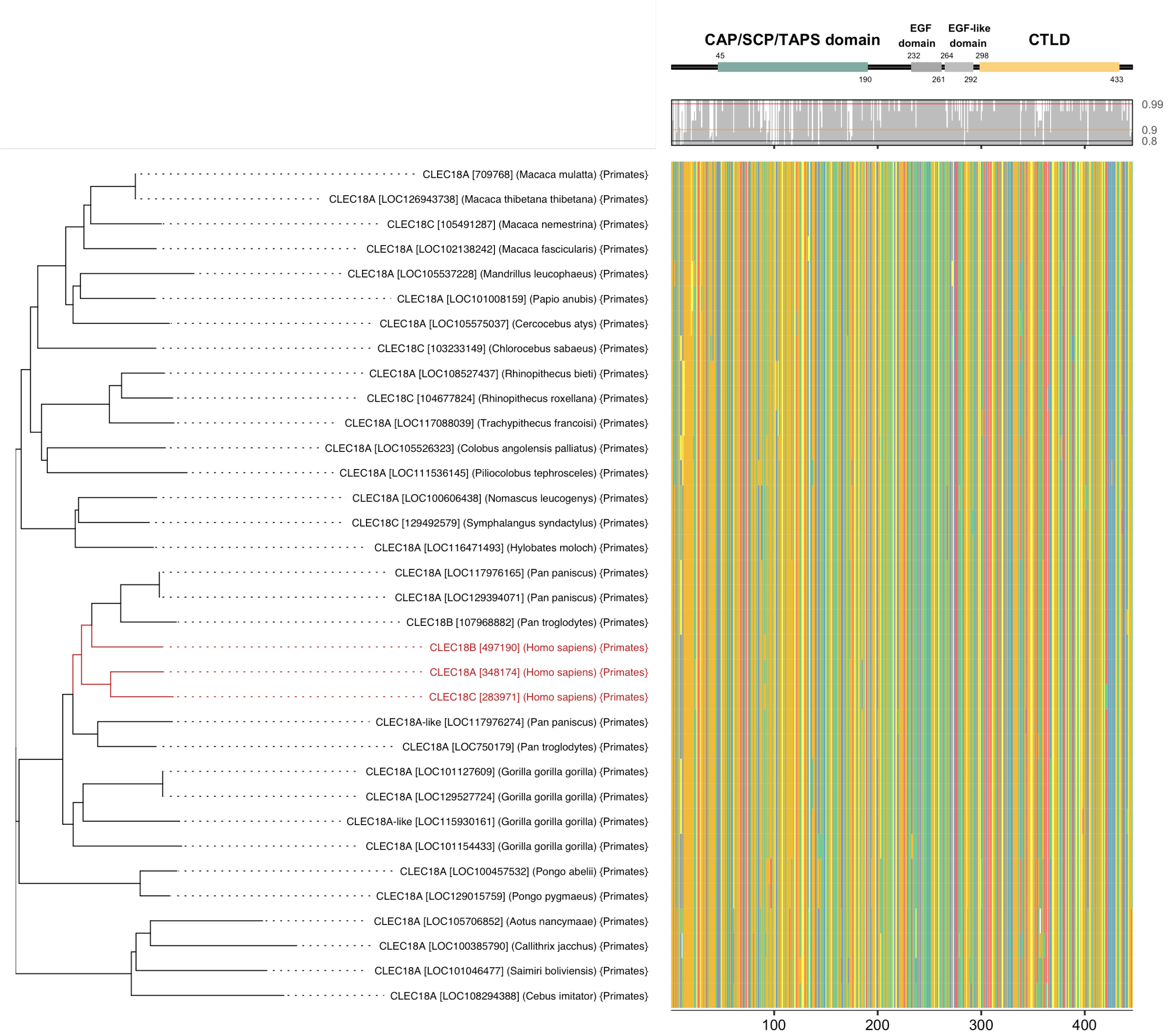


**Figure S5** Comparison of reference CLEC18 protein sequences between humans and non-human primates. Multiple sequence alignment (MSA) of CLEC18 consensus protein sequences of humans and non-human primates is shown. For humans, CLEC18 protein sequences with 446 amino acids determined by "RefSeq/MANE select" were included. As for non-human primates, predicted CLEC18 proteins of 444-448 amino acids of individual CLEC18 genes were used to determine consensus CLEC18 protein sequences. The phylogenetic tree was generated from MSAs of consensus CLEC18 protein sequences of humans and non-human primates. Sequence conservation and CLEC18 domains are shown above the MSA. Sequence conservation represents frequencies of major amino acids at individual positions across CLEC18 proteins of various species.

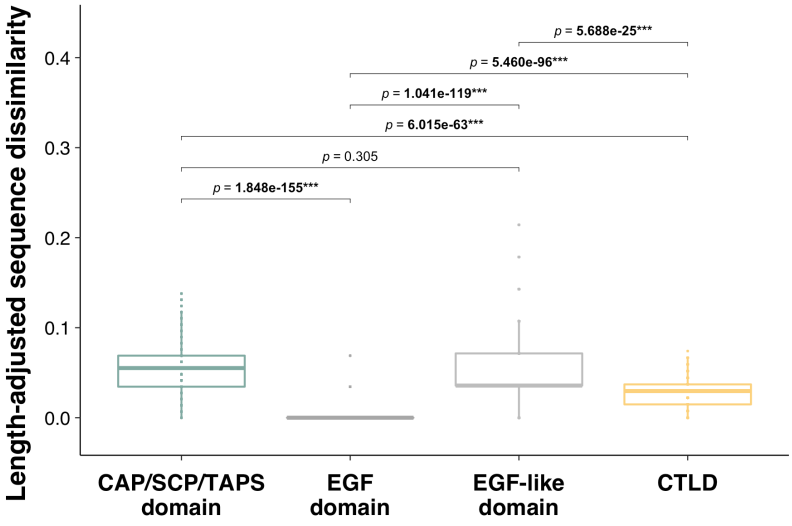


**Figure S6** Comparison of sequence dissimilarities between CLEC18 protein domains. The sequence dissimilarity was obtained from pair-wise distance matrix (Hamming distance) of CLEC18 protein domain sequences of multiple sequence alignments. The original value of sequence dissimilarity was adjusted by the amino acid length of either CAP/SCP/TAPS domain, EFG domain, EGF-like domain, or CTLD. CLEC18 domain sequences of humans and non-human primates were determined by consensus CLEC18 protein sequences of human pangenome and non-human primate reference genomes, respectively. False discovery rate (FDR)-adjusted *P*-values are shown from two-sided Wilcoxon rank-sum test, and those less than 0.05, 0.01, 0.001 are considered statistically significant and marked with "*", "**", "***", respectively.

## Supplemental Reference

1. Huang YL, Pai FS, Tsou YT, Mon HC, Hsu TL, Wu CY, Chou TY, Yang WB, Chen CH, Wong CH and Hsieh SL. Human CLEC18 Gene Cluster Contains C-type Lectins with Differential Glycan-binding Specificity. J Biol Chem. 2015;290(35):21252-21263.
